# Supplementary figures and images for: Pericentromeric heterochromatin is hierarchically organized and spatially contacts H3K9me2 islands in euchromatin
Source: PLoS Genet. 2020 Mar 23;16(3):e1008673. doi: 10.1371/journal.pgen.1008673 (PMC7147806; doi:10.1371/journal.pgen.1008673)

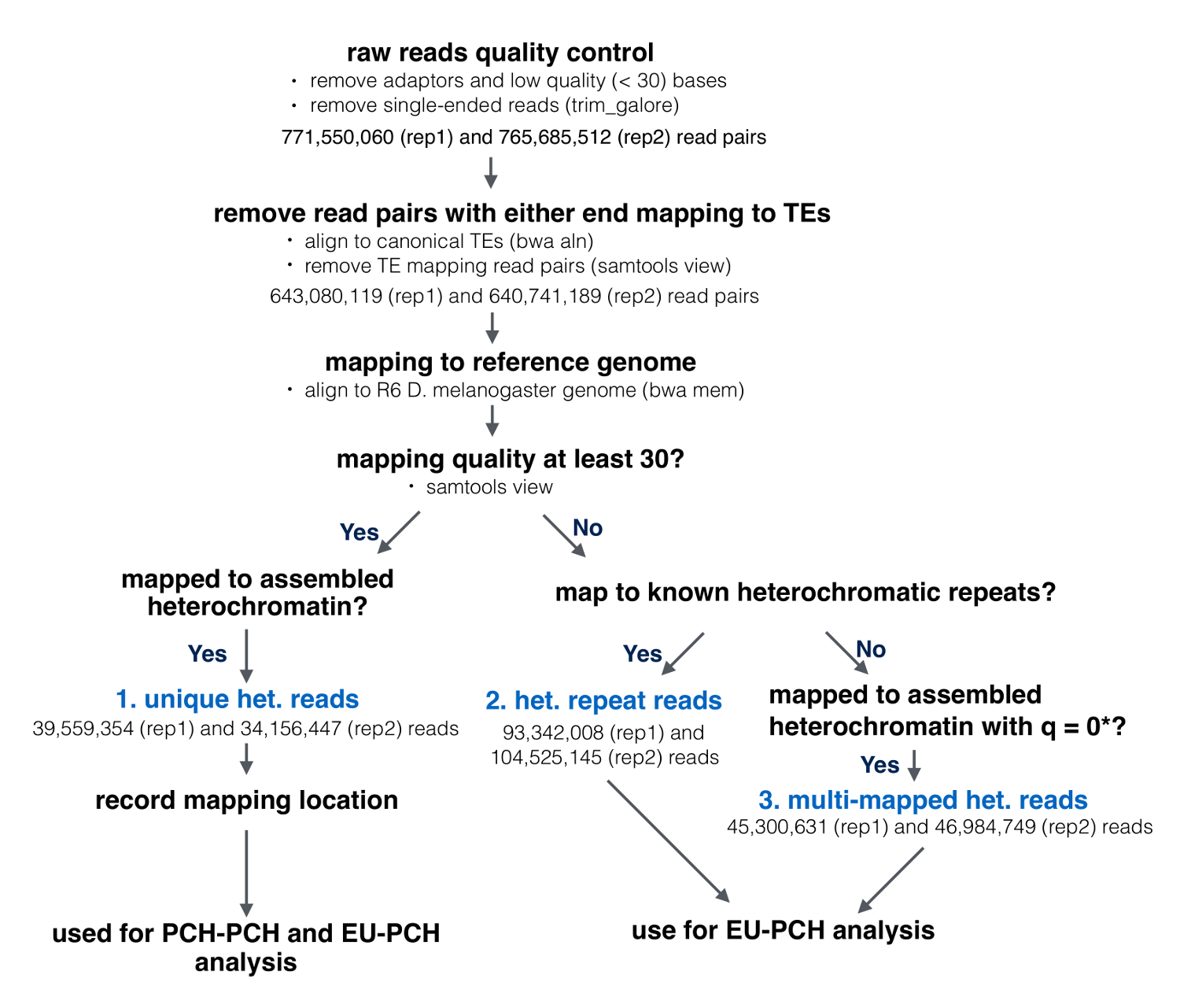

Supplement: S1 Fig — (TIFF) [file pgen.1008673.s001.tiff]

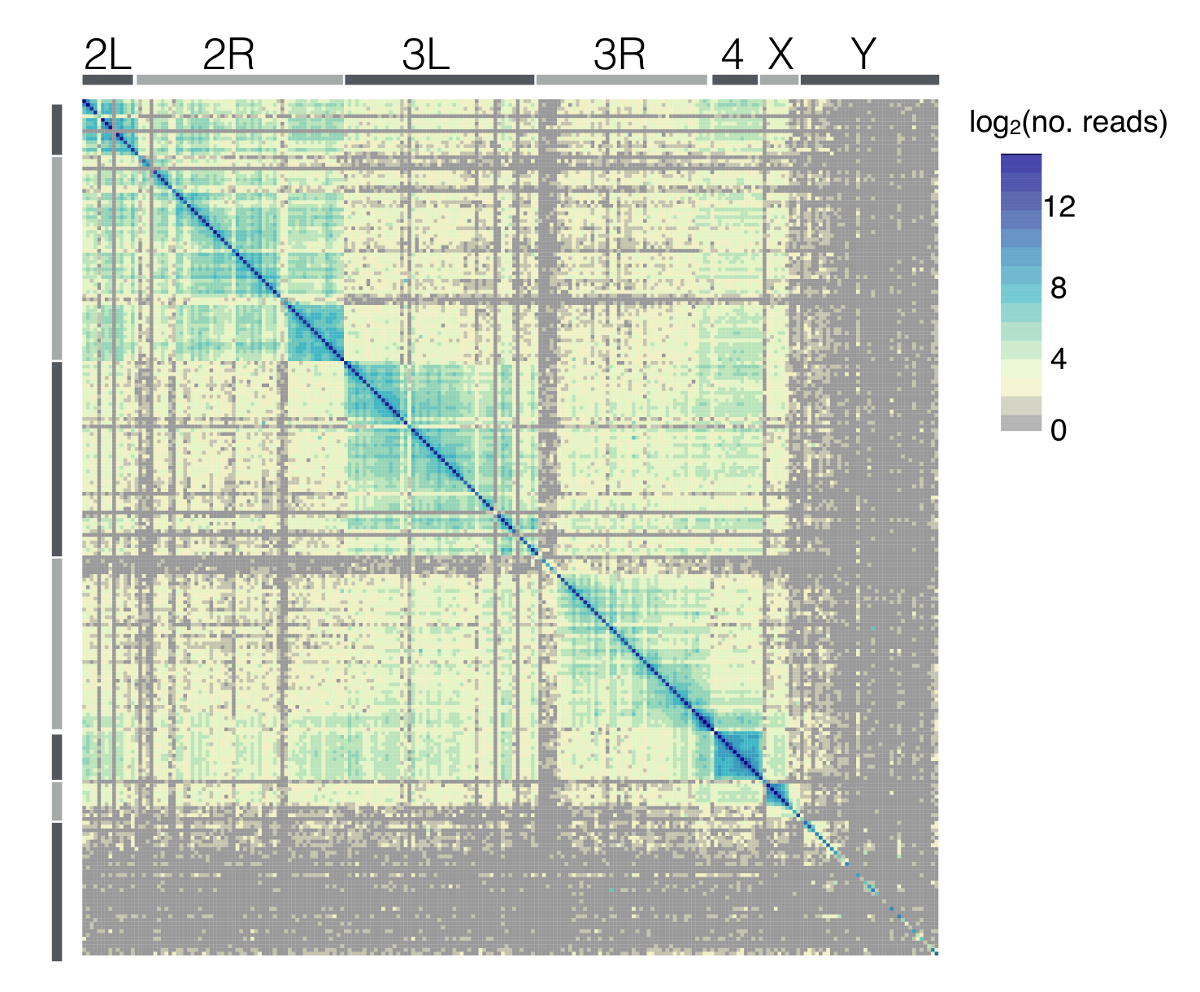

Supplement: S2 Fig — Note that only the PCH regions are shown. (TIFF) [file pgen.1008673.s002.tiff]

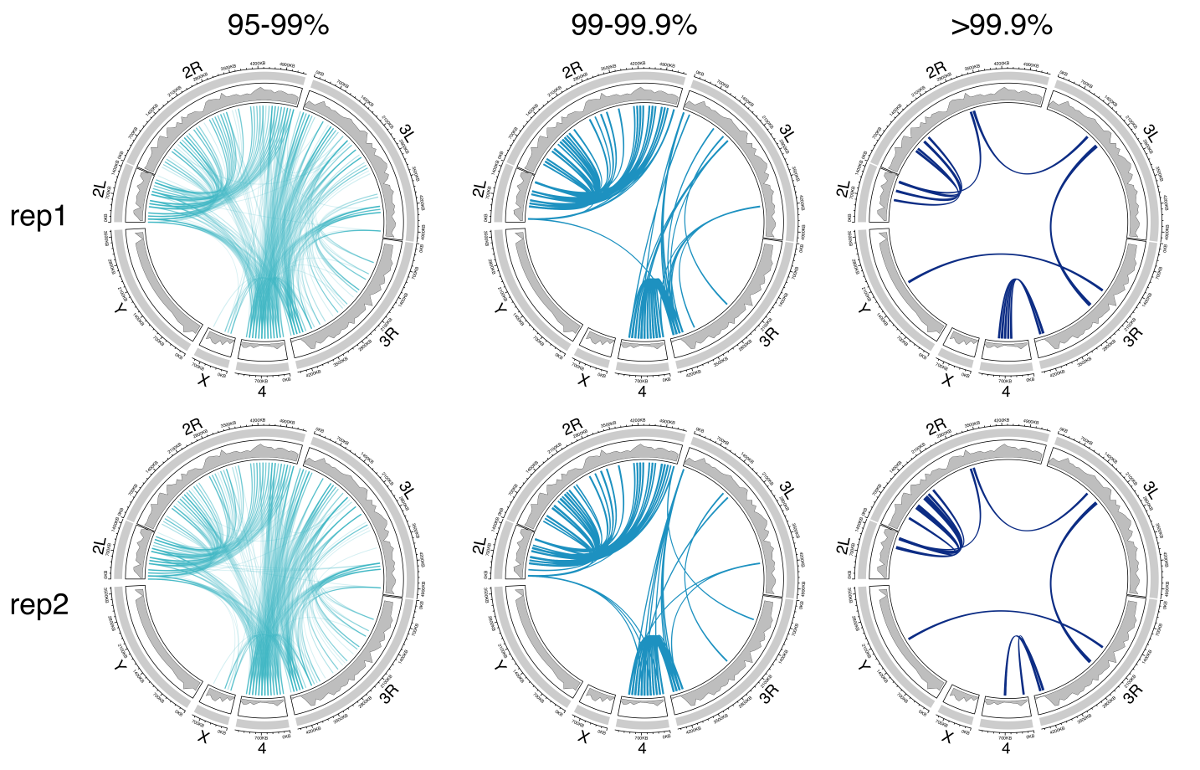

Supplement: S3 Fig — Circular plot showing inter-arm and inter-chromosomal interactions supported by 95, 99, and 99.9 percentile of Hi-C reads. Average mappability of each window is shown in the inner track. (TIFF) [file pgen.1008673.s003.tiff]

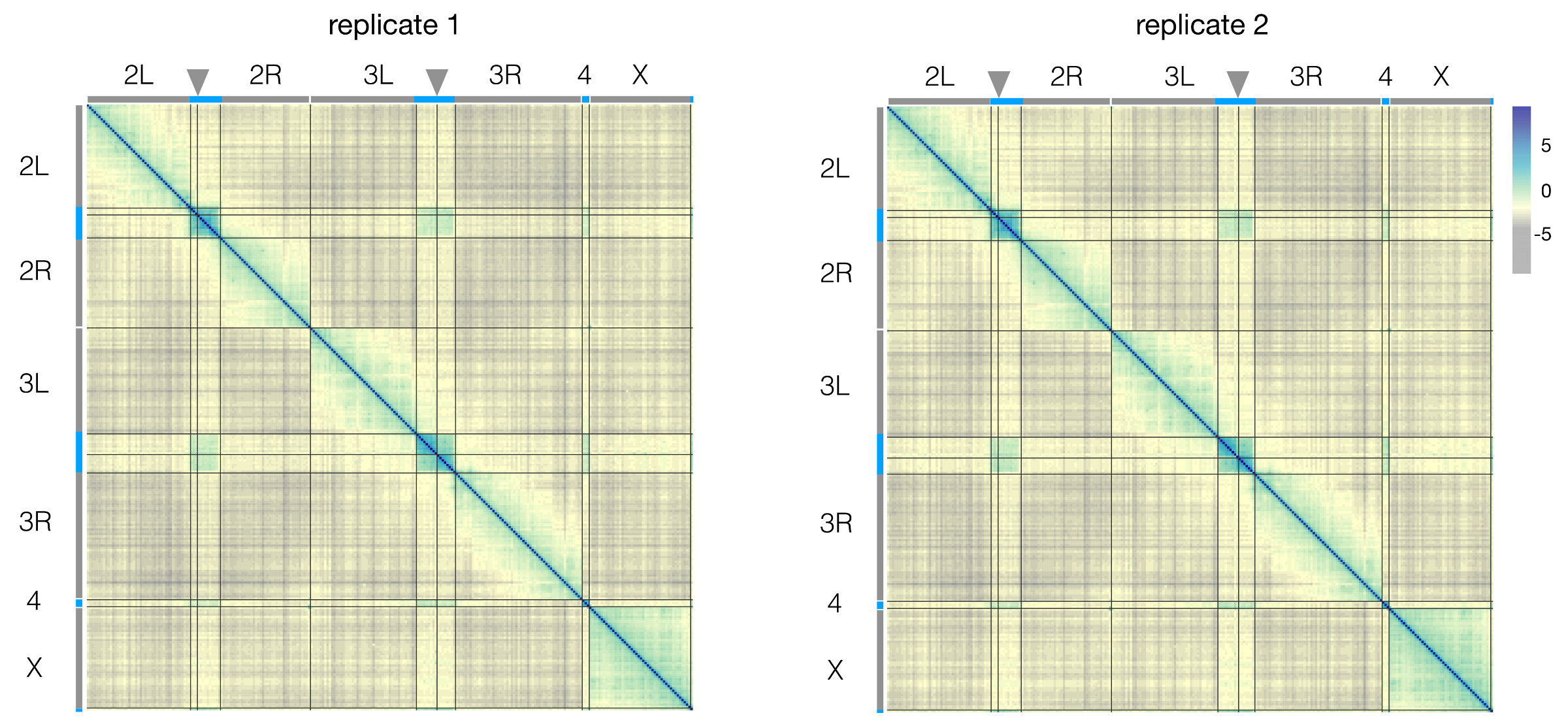

Supplement: S4 Fig — Genome-wide normalized contact map of replicate 1 (left) and replicate 2 (right). Both unique euchromatic and PCH regions are shown. Blue bars are PCH regions while gray bars are euchromatic regions. Centromeres are denoted as triangles. Each element in the matrix represents the log ratio between the number of observed contact (Hi-C read pairs) and the number of expected contacts under the assumption that each 500kb window would have equal number of total interactions across the genome. The number of observed contacts involving Y chromosome is too low for proper normalization and thus excluded from representation in the figure. Note that this normalization may be biased against interactions involving PCH regions (EU-PCH and PCH-PCH) because much fewer reads uniquely mapped to PCH regions than euchromatic regions. (TIFF) [file pgen.1008673.s004.tiff]

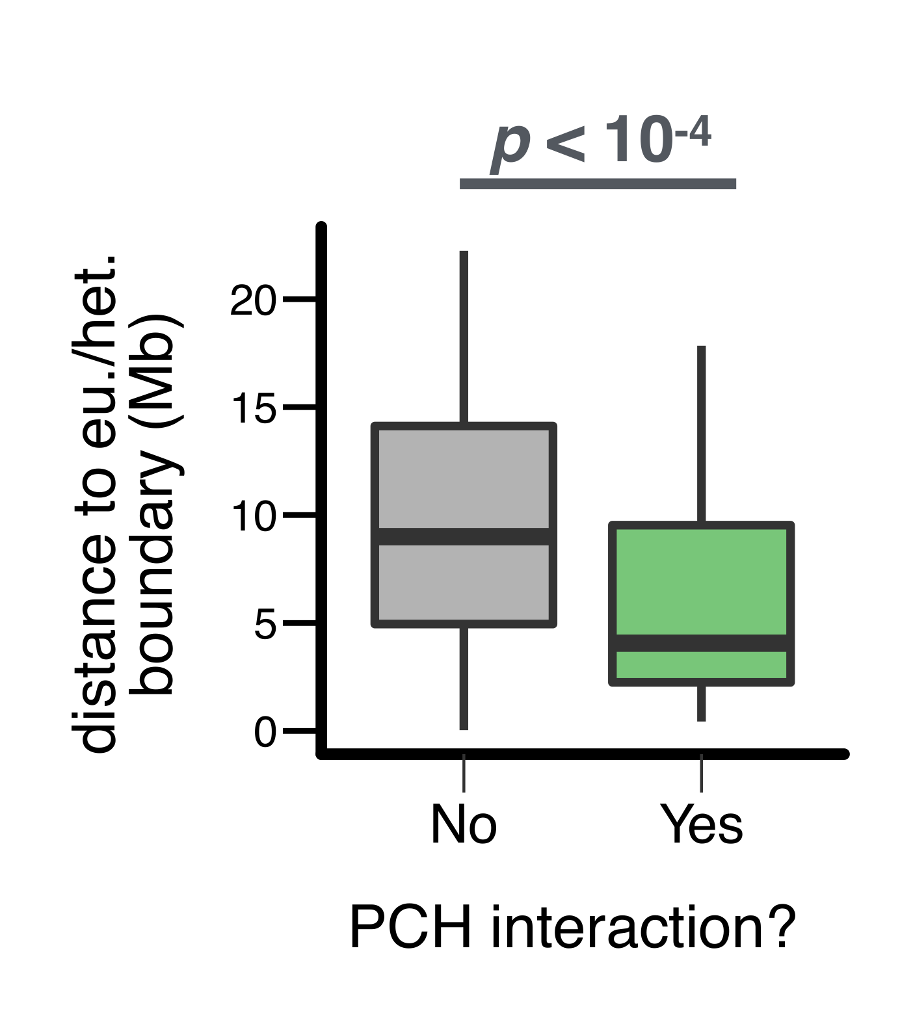

Supplement: S5 Fig — H3K9me2-enriched with and without PCH interactions are in green and gray respectively. (TIFF) [file pgen.1008673.s005.tiff]

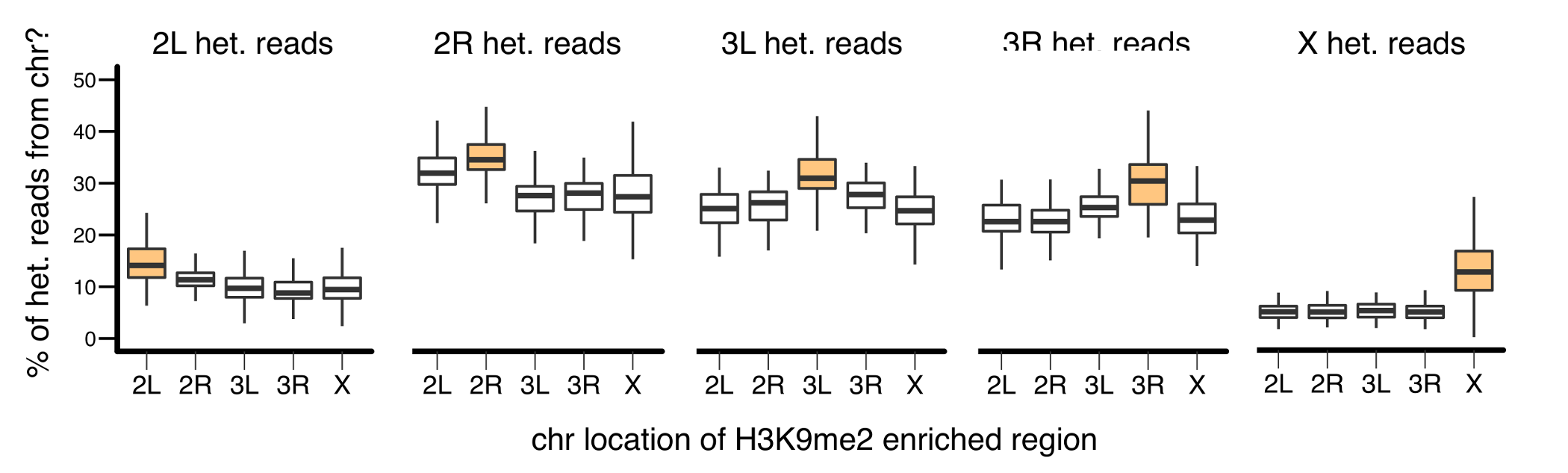

Supplement: S6 Fig — Data for replicate 2 is shown. (TIFF) [file pgen.1008673.s006.tiff]

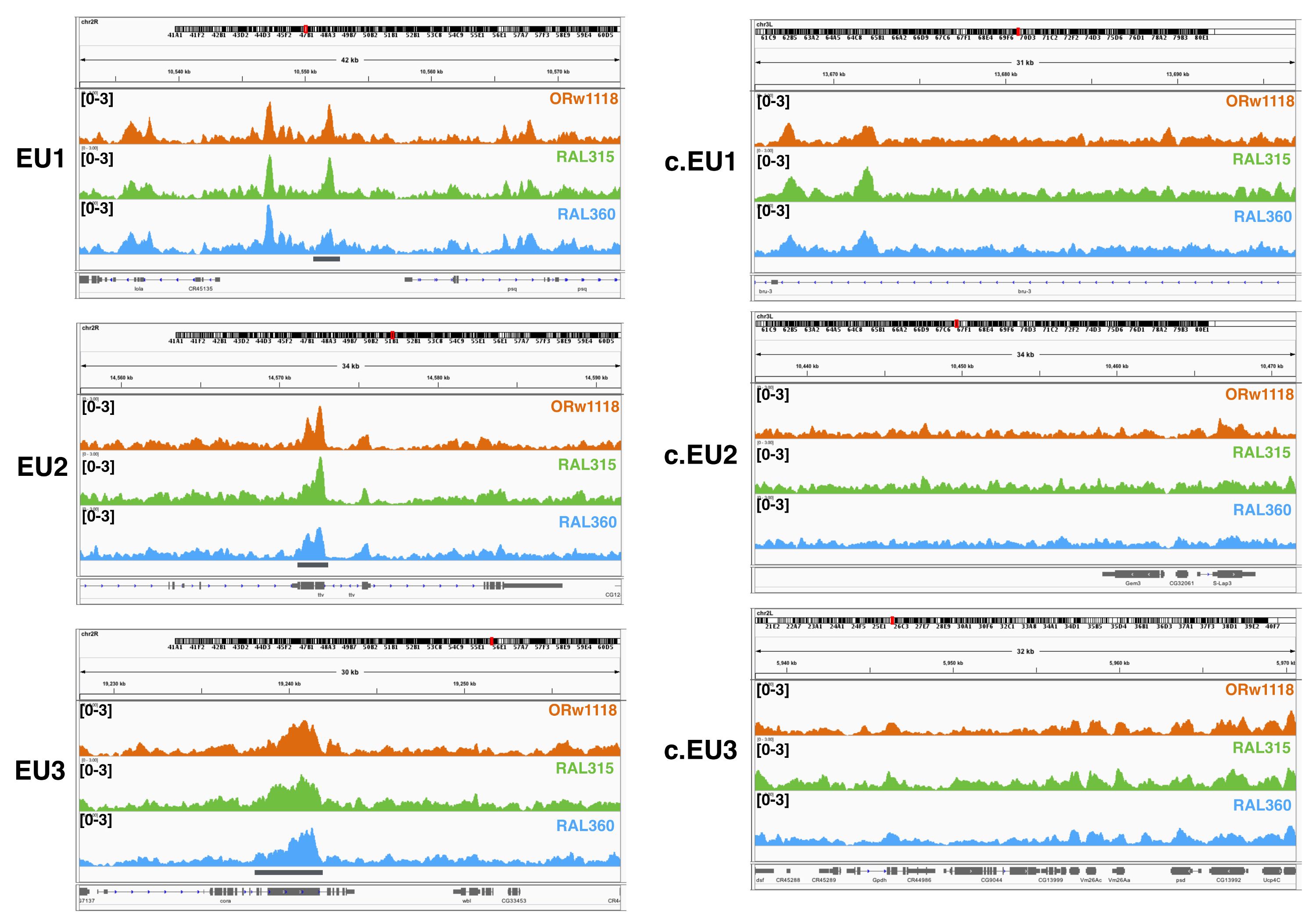

Supplement: S7 Fig — There is H3K9me2 enrichment in both ORw1118 and wildtype strains for EU1-3, but none for control regions c.EU1-3. The fourth tracks (below RAL360, blue) are broad peaks called by Macs2 in ORw1118. (TIFF) [file pgen.1008673.s007.tiff]

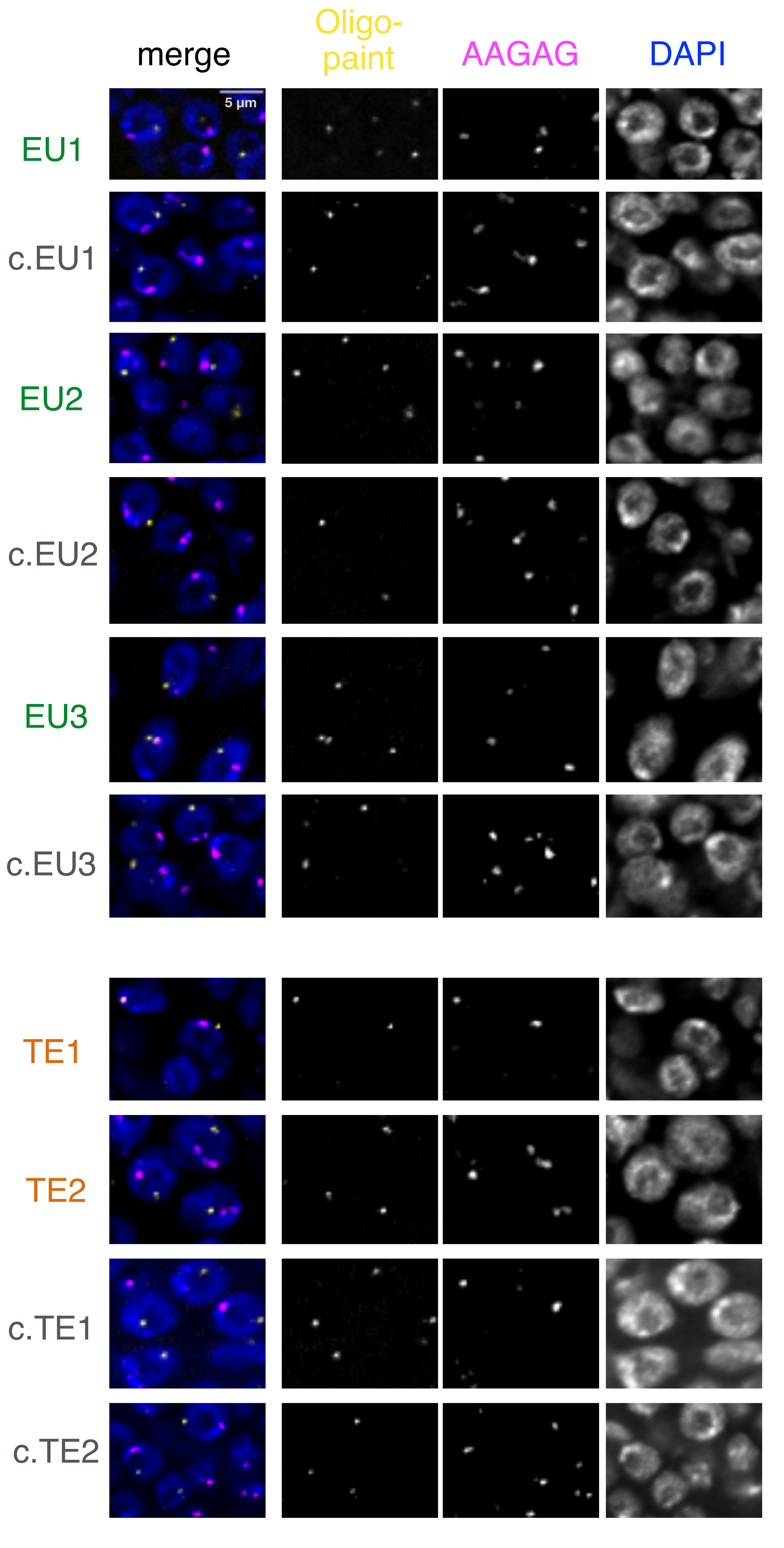

Supplement: S8 Fig — (TIFF) [file pgen.1008673.s008.tiff]

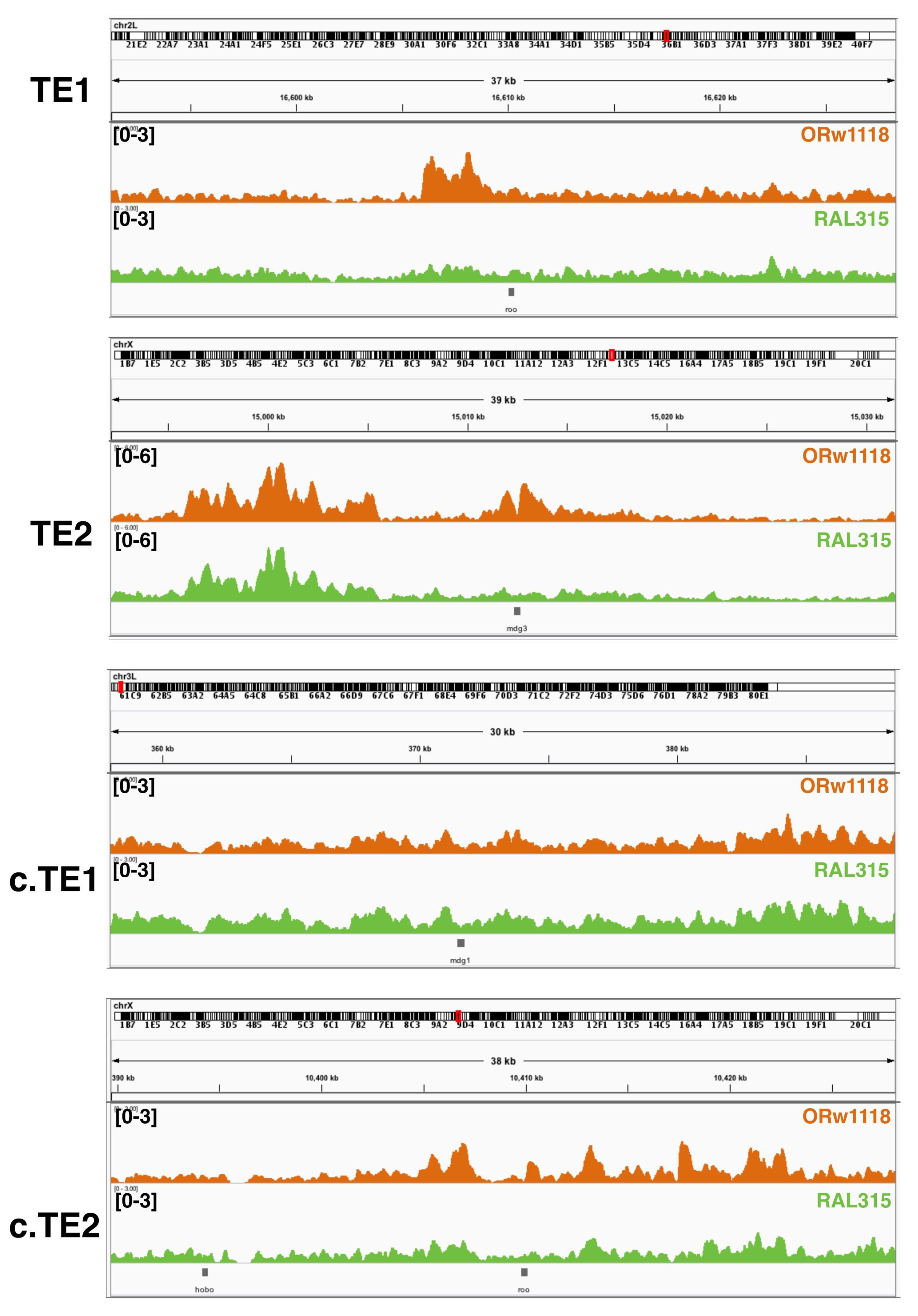

Supplement: S9 Fig — Strain-specific H3K9me2 enrichment was observed for TE1 and TE2. Third track (one below RAL315, green) shows the insertion position of TEs in ORw1118 identified by TIDAL. (TIFF) [file pgen.1008673.s009.tiff]

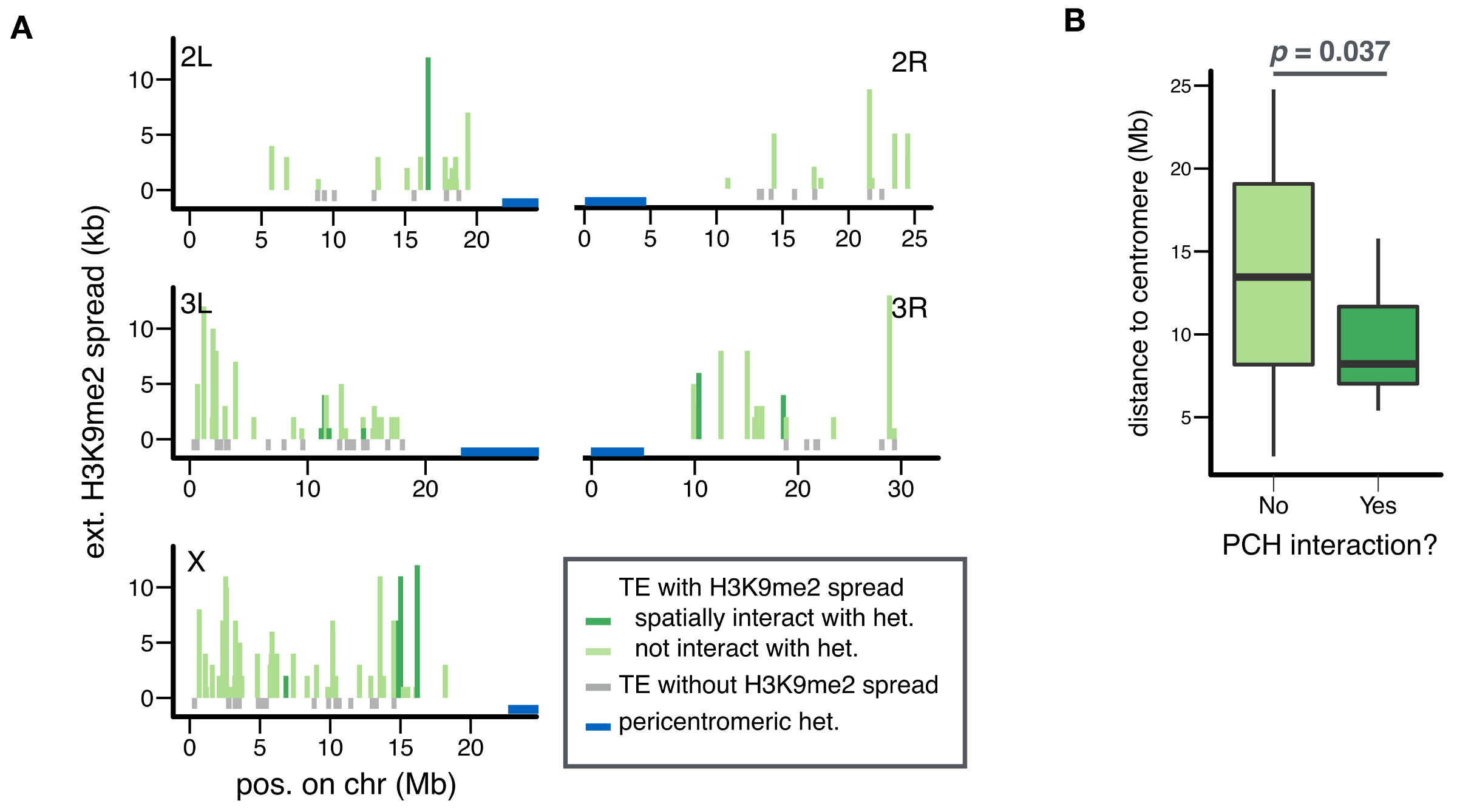

Supplement: S10 Fig — (A) The extent of local H3K9me2 enrichment at TEs is shown on the y-axis for TEs with (green) and without (gray) local H3K9me2 enrichment, and with (dark green) and without (light green) PCH interaction. (B) The linear distance between PCH and TEs with (dark green) and without (light green) PCH interactions are shown in boxplots. (TIFF) [file pgen.1008673.s010.tiff]

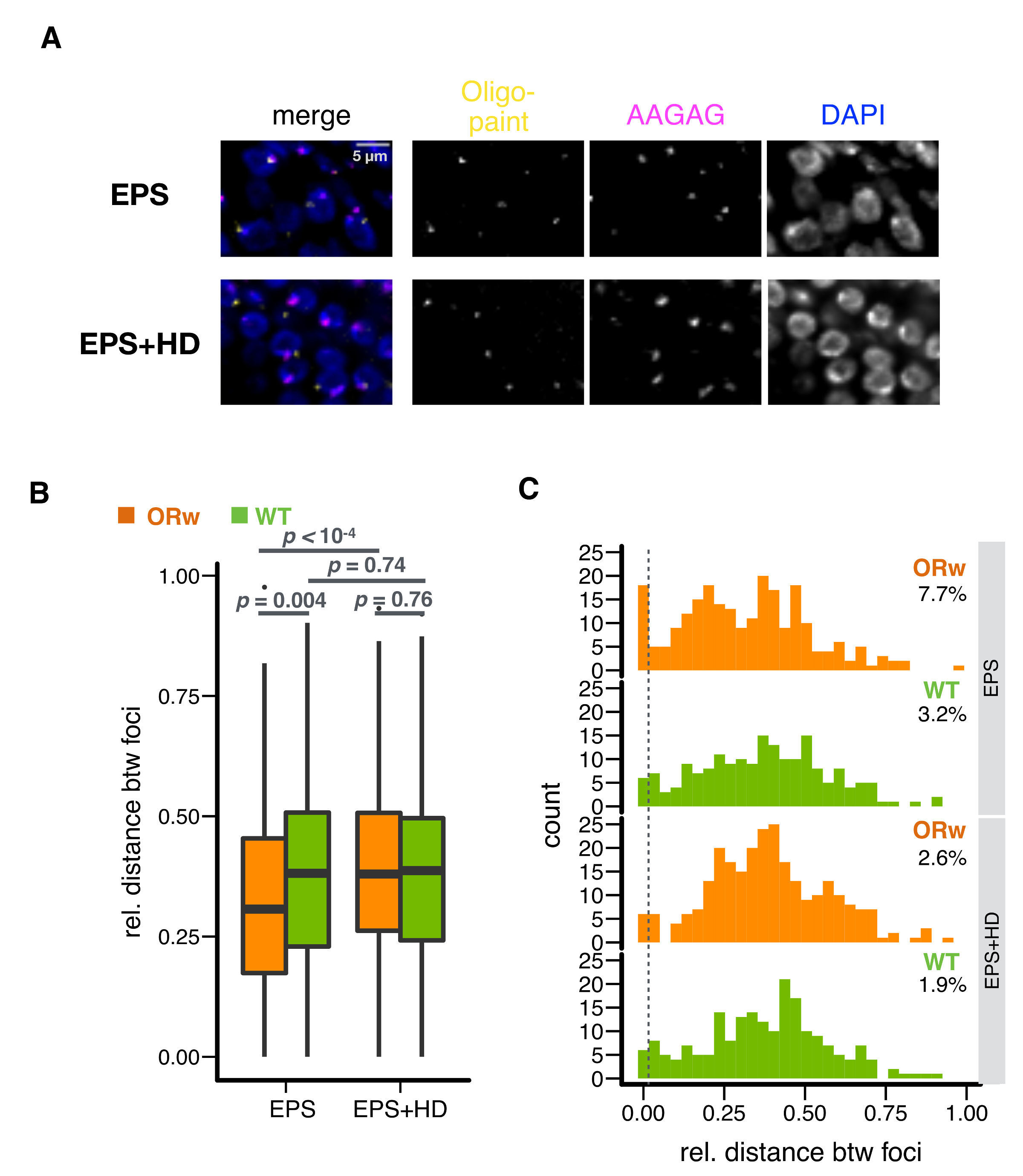

Supplement: S11 Fig — (A) Representative FISH images for permeabilized embryos (EPS) and permeabilized embryos with 1,6-hexanediol treatment (EPS+HD). (B) Boxplot and (C) histogram showing the relative distance between TE1 and PCH. Comparisons of the distance between pairs of foci were tested with Mann-Whitney test (p-values in (A)) and Fisher’s exact test (for proportion of overlapping foci, p-values = 0.02 (ORw, EPS vs. EPS+HD), 1 (WT, EPS vs. EPS+HD), 0.057 (ESP treatment, ORw vs. WT), 0.55 (HD treatment, ORw vs. WT)). Threshold for nuclei with overlapping foci is denoted with a dashed line. (TIFF) [file pgen.1008673.s011.tiff]

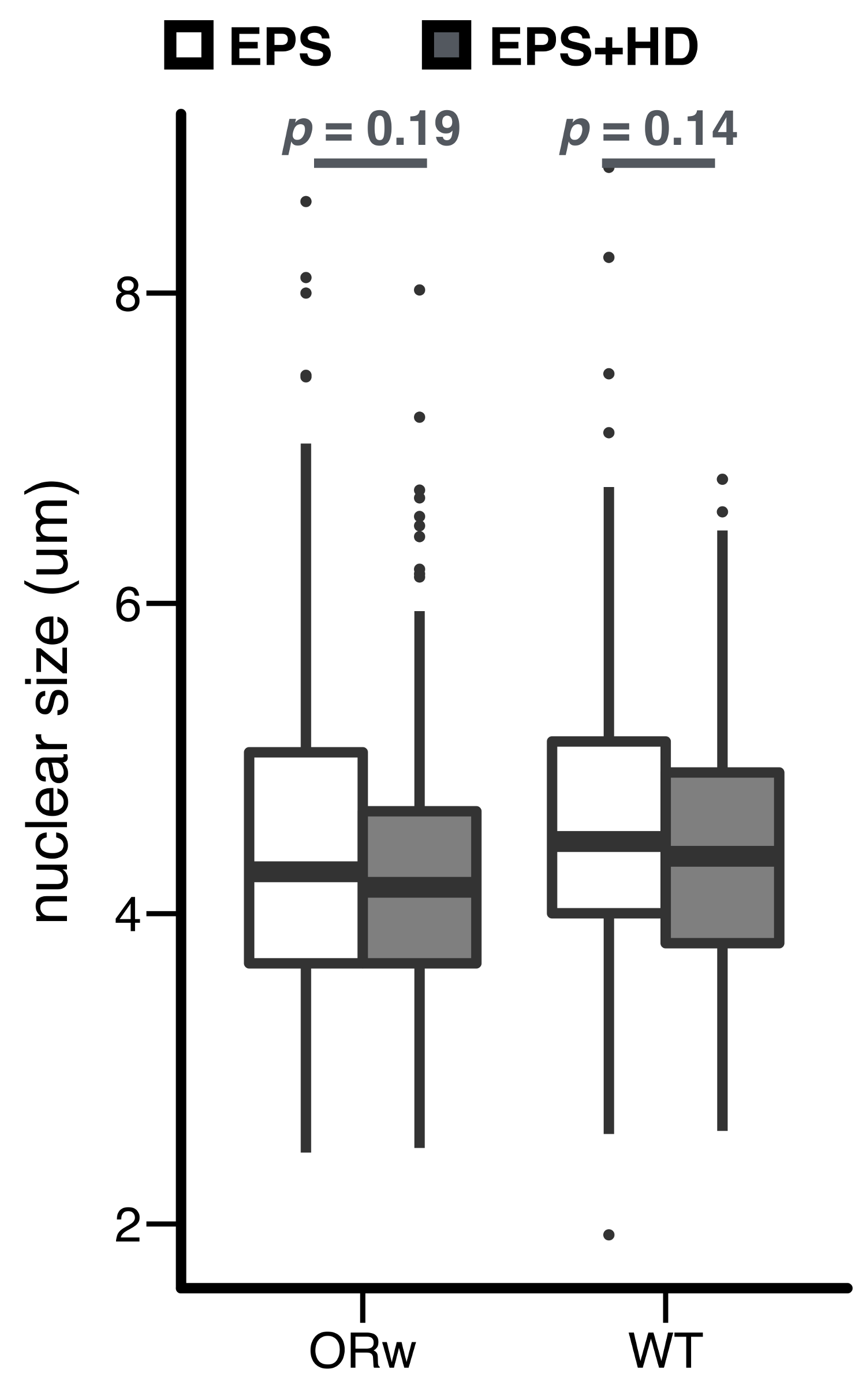

Supplement: S12 Fig — EPS: permeabilized embryos; EPS+HD: permeabilized embryos with 1,6-hexanediol treatments. (TIFF) [file pgen.1008673.s012.tiff]

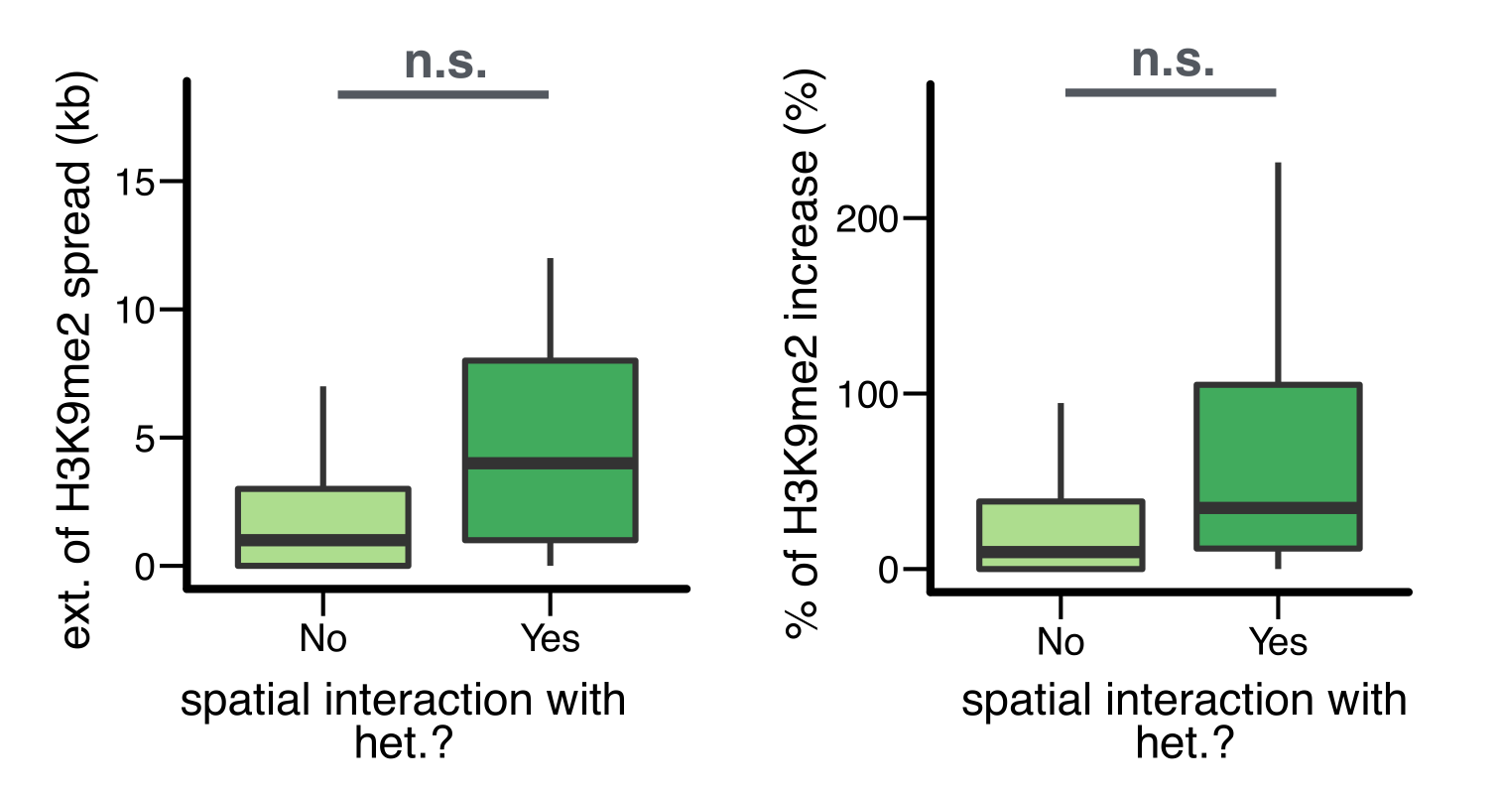

Supplement: S13 Fig — (TIFF) [file pgen.1008673.s013.tiff]

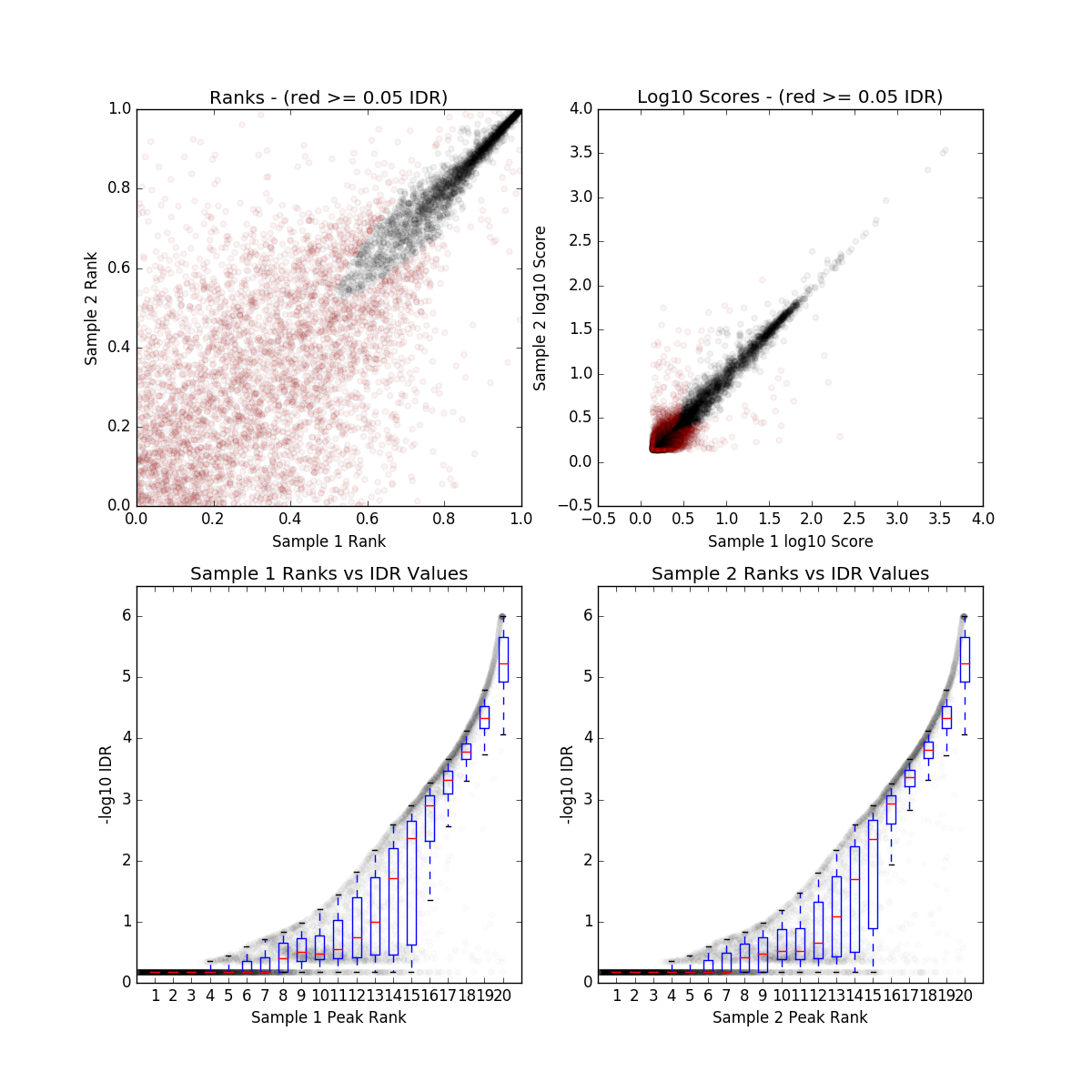

Supplement: S14 Fig — (TIF) [file pgen.1008673.s014.tif]

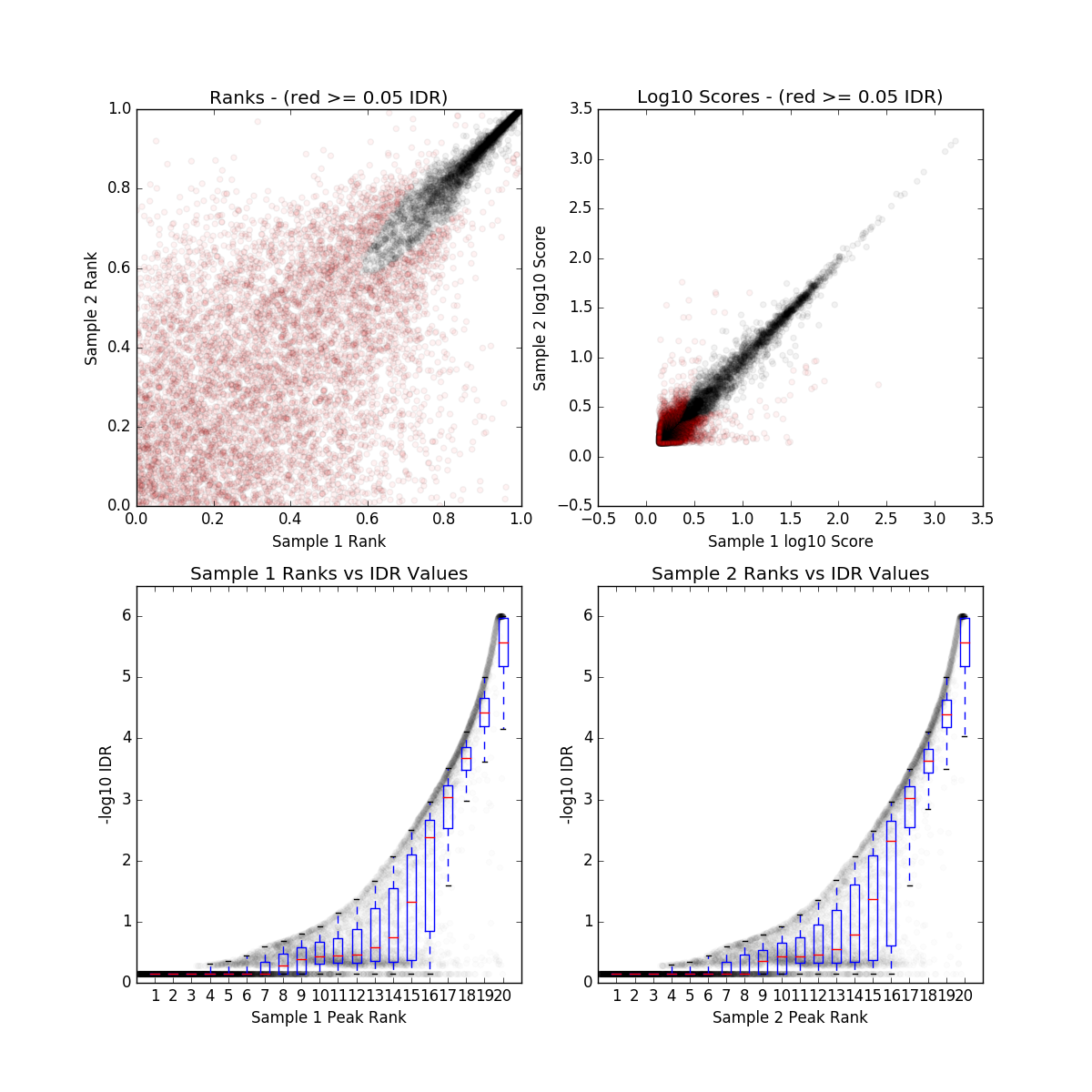

Supplement: S15 Fig — (TIF) [file pgen.1008673.s015.tif]

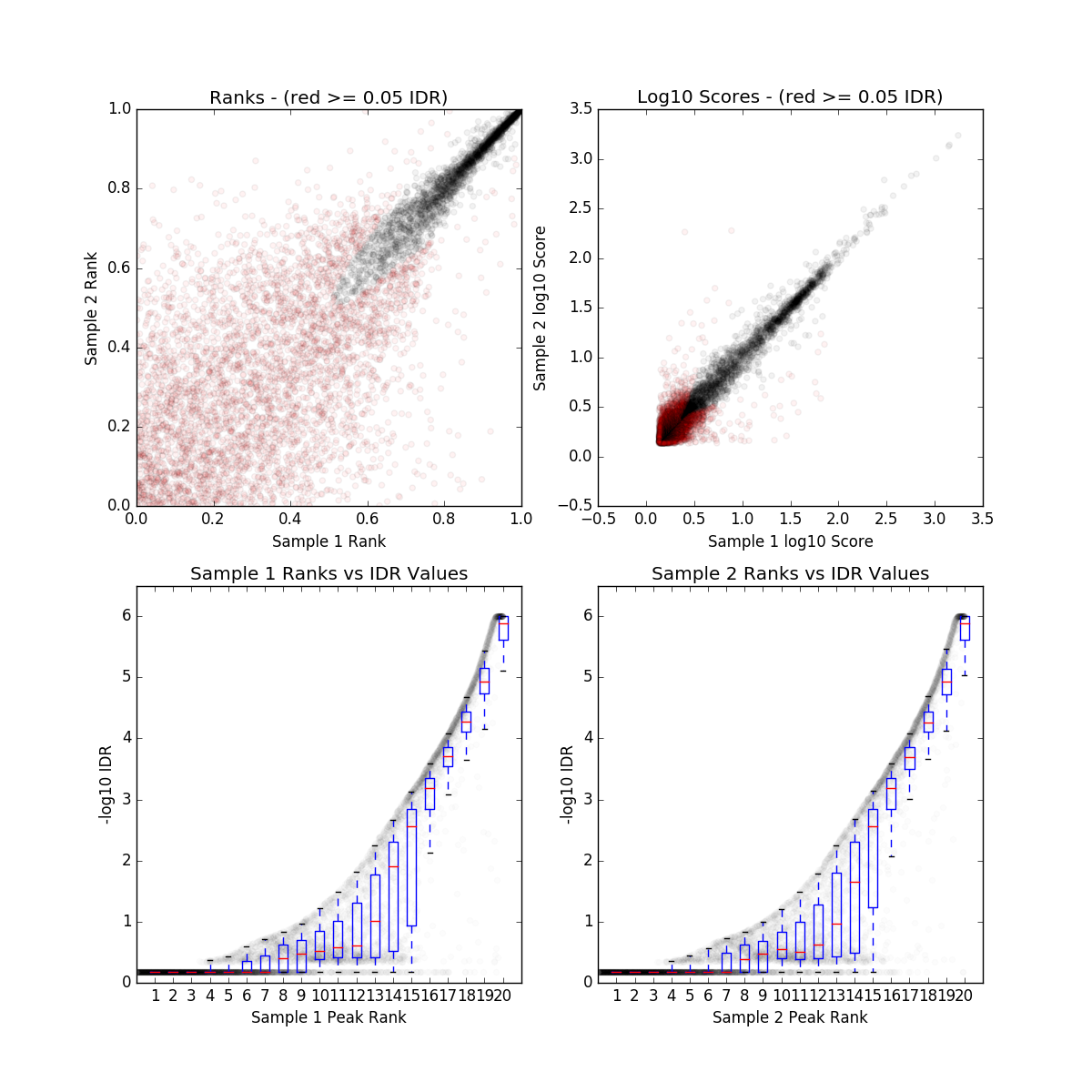

Supplement: S16 Fig — (TIF) [file pgen.1008673.s016.tif]

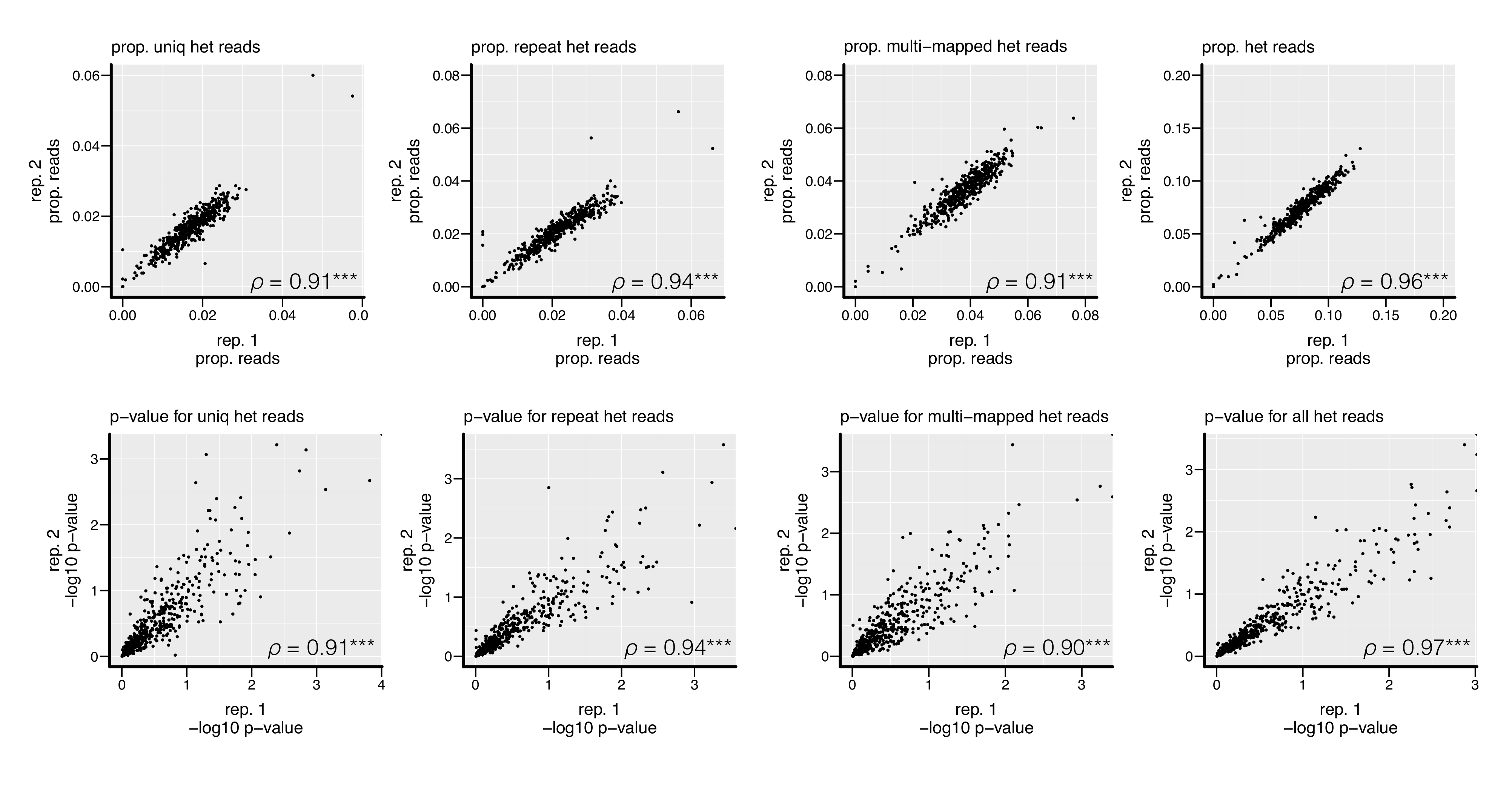

Supplement: S17 Fig — ***p < 0.001. (TIFF) [file pgen.1008673.s017.tiff]

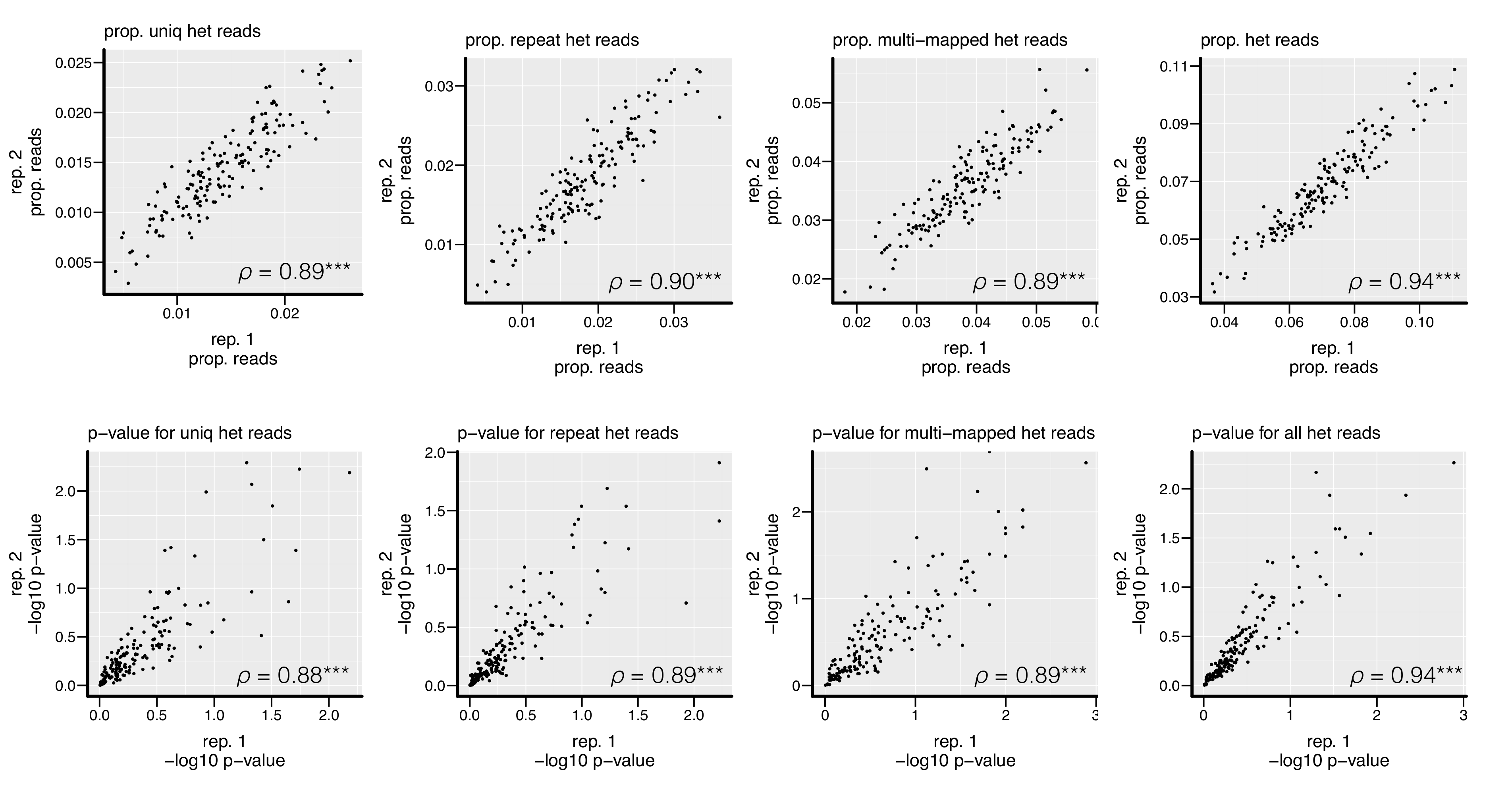

Supplement: S18 Fig — ***p < 0.001. (TIFF) [file pgen.1008673.s018.tiff]

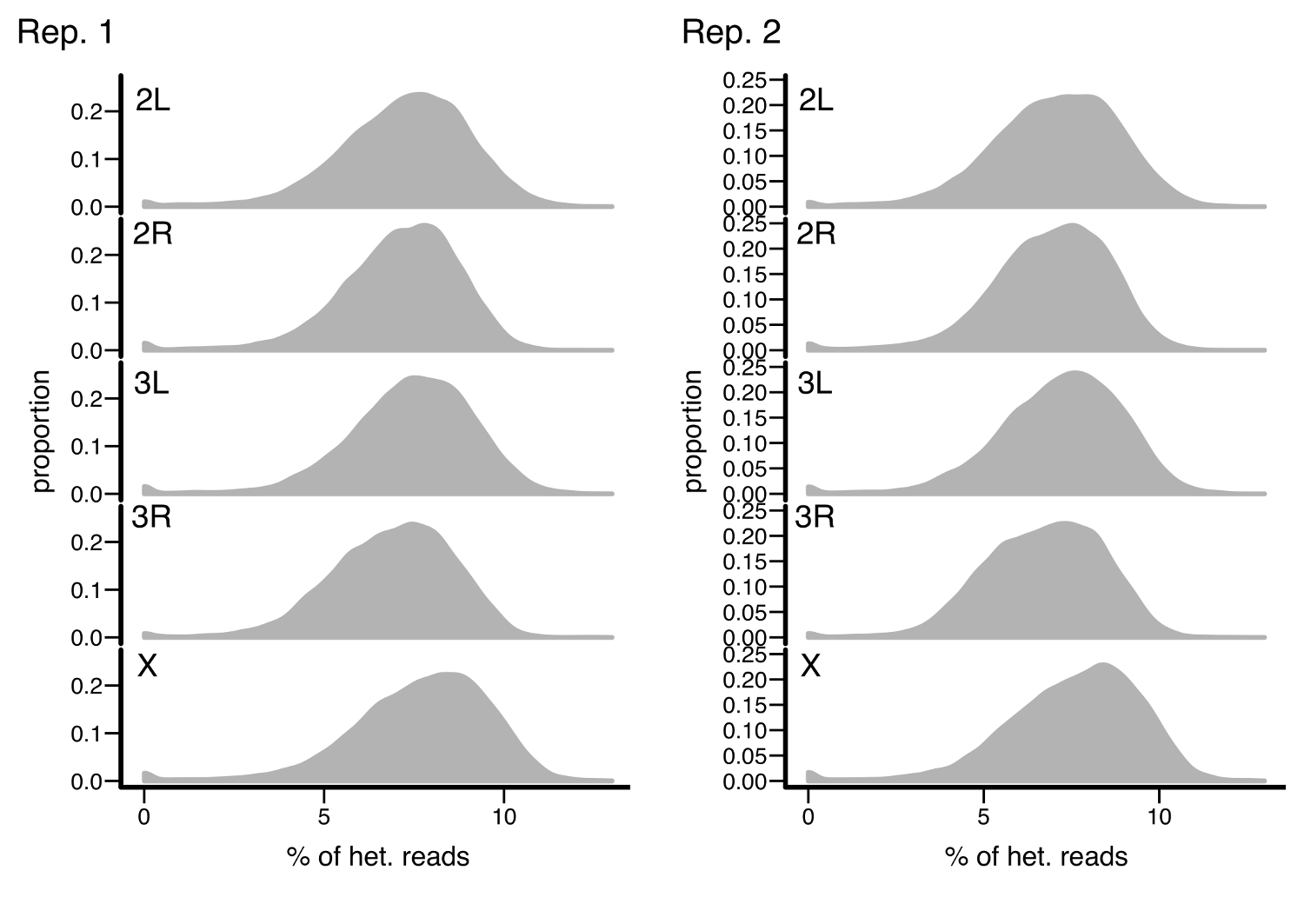

Supplement: S19 Fig — (TIFF) [file pgen.1008673.s019.tiff]

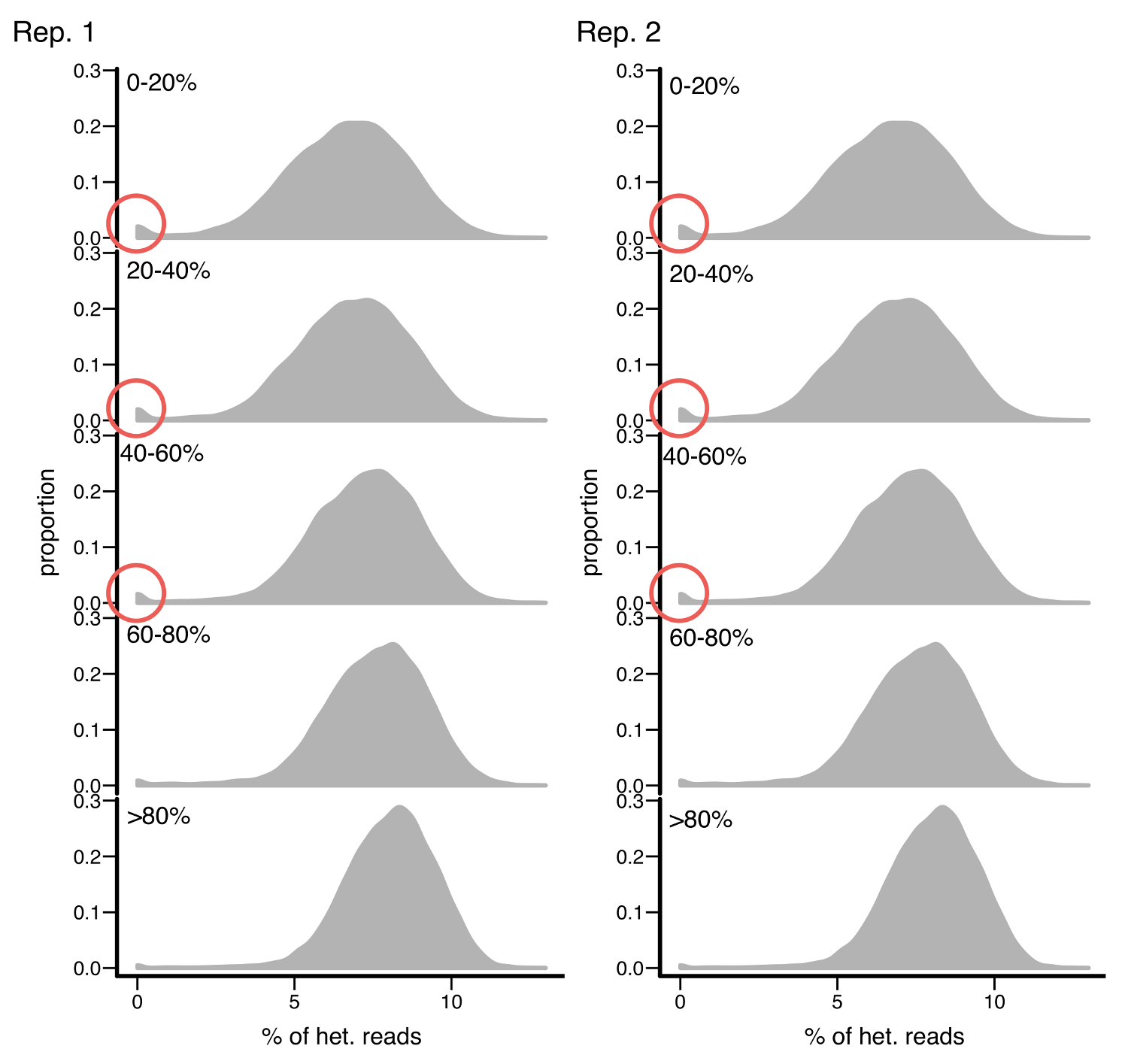

Supplement: S20 Fig — (TIFF) [file pgen.1008673.s020.tiff]
