# Supplementary material for: Pericentromeric heterochromatin is hierarchically organized and spatially contacts H3K9me2 islands in euchromatin
Source: PLoS Genet. 2020 Mar 23;16(3):e1008673. doi: 10.1371/journal.pgen.1008673 (PMC7147806; doi:10.1371/journal.pgen.1008673)
Supplement: S1 Table — (PDF) [file pgen.1008673.s021.pdf]

S1 Table. List of heterochromatic simple and complex repeats

Repeat	Note
1.686	
1.688	
AAAACAT	
AAAAG	
AAAATAT	AATAT variant
AAACAAT	
AAACAC	AACAC variant
AAAGAC	AAGAC variant
AAATTACT	
AACAC	1.672
AAGAC	1.689, 1.701
AAGACATGAC	
AAGACTAGAC	
AAGAG	1.705
AAGAGAAGAGAG	AAGAG variant
AAGAGAG	AAGAG variant
AAGAGG	AAGAG variant
AAGGAG	AAGAG variant
AATAC	1.68
AATAG	1.693
AATAGAC	1.688
AATAT	1.672
AATATAT	AATAT variant
ACATATAT	
ACCAGTACGGG	undeca
ACCGAGTACGGG	dodeca
AGATG	
His1	
His2A	
His2B	
His3	
His4	
rDNA	
Rsp	
