# Supplementary material for: Pericentromeric heterochromatin is hierarchically organized and spatially contacts H3K9me2 islands in euchromatin
Source: PLoS Genet. 2020 Mar 23;16(3):e1008673. doi: 10.1371/journal.pgen.1008673 (PMC7147806; doi:10.1371/journal.pgen.1008673)
Supplement: S2 Table — (PDF) [file pgen.1008673.s022.pdf]

S2 Table. Number of Hi-C read pairs for pairs of PCH regions

		no. of read pairs		% of all read pairs	
chr 1	chr 2	**replicate 1**	**replicate 2**	**replicate 1**	**replicate 2**
2L	2L	1164391	773042	9.66%	9.63%
2L	2R	37392	38783	0.31%	0.48%
2L	3L	9525	9277	0.08%	0.12%
2L	3R	7190	6802	0.06%	0.08%
2L	4	6822	6949	0.06%	0.09%
2L	X	1385	1408	0.01%	0.02%
2L	Y	421	452	0.00%	0.01%
2R	2R	2758050	1778783	22.87%	22.15%
2R	3L	26163	24688	0.22%	0.31%
2R	3R	20945	20818	0.17%	0.26%
2R	4	19009	18245	0.16%	0.23%
2R	X	4303	4177	0.04%	0.05%
2R	Y	1491	1472	0.01%	0.02%
3L	3L	2490763	1600901	20.66%	19.94%
3L	3R	42885	43355	0.36%	0.54%
3L	4	22377	21738	0.19%	0.27%
3L	X	3526	3532	0.03%	0.04%
3L	Y	1432	1416	0.01%	0.02%
3R	3R	2492544	1731036	20.67%	21.56%
3R	4	17962	17364	0.15%	0.22%
3R	X	3348	3251	0.03%	0.04%
3R	Y	1649	1765	0.01%	0.02%
4	4	2050167	1321436	17.00%	16.46%
4	X	2414	2552	0.02%	0.03%
4	Y	771	678	0.01%	0.01%
X	X	778807	537628	6.46%	6.69%
X	Y	229	231	0.00%	0.00%
Y	Y	91377	58732	0.76%	0.73%
