# Supplementary material for: Pericentromeric heterochromatin is hierarchically organized and spatially contacts H3K9me2 islands in euchromatin
Source: PLoS Genet. 2020 Mar 23;16(3):e1008673. doi: 10.1371/journal.pgen.1008673 (PMC7147806; doi:10.1371/journal.pgen.1008673)
Supplement: S3 Table — (PDF) [file pgen.1008673.s023.pdf]

S3 Table. Chromatin environment of euchromatic H3K9me2-enriched regions interacting with PCH

Sexton et al. 2012					Active vs other		Null vs other	
3D interaction with PCH	Active	HP1a-enriched	Null	Polycomb-enriched	FET *p-value*	odds ratio	FET *p-value*	odds ratio
Yes	18	1	20	4	7.76E-03	2.51	3.05E-02	0.48
No	100	3	291	57				

Filon et al. 2010						Red+Yellow vs other		Black vs other	
3D interaction with PCH	BLACK	BLUE	GREEN	RED	YELLOW	FET *p-value*	odds ratio	FET *p-value*	odds ratio
Yes	12	12	2	0	16	2.10E-02	2.22	5.19E-04	0.30
No	260	82	13	17	81				

Kharchenko et al. 2011										1,2,3,4 vs other		9 vs other	
3D interaction with PCH	1	2	3	4	5	6	7	8	9	FET *p-value*	odds ratio	FET *p-value*	odds ratio
**S2 cells**													
Yes	4	5	1	3	5	1	0	0	5	5.27E-05	6.14	8.64E-03	0.26
No	9	19	3	19	34	8	3	11	106				
**BG3 cells**													
Yes	2	4	2	3	4	0	0	5	5	1.13E-02	3.05	7.85E-01	1.16
No	10	9	13	21	22	11	3	125	46				
