# Supplementary material for: Pericentromeric heterochromatin is hierarchically organized and spatially contacts H3K9me2 islands in euchromatin
Source: PLoS Genet. 2020 Mar 23;16(3):e1008673. doi: 10.1371/journal.pgen.1008673 (PMC7147806; doi:10.1371/journal.pgen.1008673)
Supplement: S4 Table — (PDF) [file pgen.1008673.s024.pdf]

S4 Table. Properties of euchromatic H3K9me2-enriched regions interacting with PCH

	**3D interaction criteria**			
	**both rep. sig.** *p-values*	**either rep.sig** *p-values*	**test type**	**Note**
Distance to centromere	**5.0E-02**	**2.0E-04**	*Mann-Whitney U*	with 3D interaction shorter
proportion of coding sequence	**4.7E-03**	**7.6E-04**	*Mann-Whitney U*	with 3D interaction higher
X vs autosome	3.1E-01	2.7E-01	*Fisher's Exact*	
size of H3K9me2 enriched region	3.4E-01	3.1E-01	*Mann-Whitney U*	
H3K9me2 enrichment	9.5E-01	9.1E-01	*Mann-Whitney U*	
enrichment of Active TAD (Sexton et al. 2012)	**7.8E-03**	**3.2E-02**	*Fisher's Exact*	odds ratio = 1.88 (either)
enrichment of Red and Yellow chromatin (Filion et al. 2010)	**2.1E-02**	1.2E-01	*Fisher's Exact*	odds ratio = 1.59 (either)
enrichment of 1-4 of modEncode 9 states, S2 (Kharchenko et al. 2011)	**5.3E-05**	**1.6E-02**	*Fisher's Exact*	odds ratio = 2.55 (either)
enrichment of 1-4 of modEncode 9 states, BG3 (Kharchenko et al. 2011)	**1.1E-02**	**3.8E-02**	*Fisher's Exact*	odds ratio = 2.20 (either)
depletion of Null TAD (Sexton et al. 2012)	**3.1E-02**	**1.0E-02**	*Fisher's Exact*	odds ratio = 0.50 (either)
depletion of Black chromatin (Filion et al. 2010)	**5.2E-04**	**6.0E-04**	*Fisher's Exact*	odds ratio = 0.40 (either)
depletion of 9 of modEncode 9 states, S2 (Kharchenko et al. 2011)	**8.6E-03**	**7.3E-03**	*Fisher's Exact*	odds ratio = 0.35 (either)
depletion of 9 of modEncode 9 states, BG3 (Kharchenko et al. 2011)	7.9E-01	3.8E-01	*Fisher's Exact*	
