# Supplementary material for: Pericentromeric heterochromatin is hierarchically organized and spatially contacts H3K9me2 islands in euchromatin
Source: PLoS Genet. 2020 Mar 23;16(3):e1008673. doi: 10.1371/journal.pgen.1008673 (PMC7147806; doi:10.1371/journal.pgen.1008673)
Supplement: S5 Table — (PDF) [file pgen.1008673.s025.pdf]

S5 Table. Information for regions targeted by Oligopaint

ID	chr	window start	window end	no. probes	probe density (per kb)	distance to centromere (Mb)	Notes
TE1	2L	16591247	16628353	504	13.58	2.96	roo
TE2	X	14992136	15031508	505	12.83	3.92	mdg3
c.TE1	3L	357967	388478	507	16.62	18.07	mdg1
c.TE2	X	10389680	10428155	507	13.18	8.52	roo
EU1	2R	10532124	10575010	503	11.73	1.69	
EU2	2R	14557395	14591631	503	14.69	5.71	
EU3	2R	19228022	19258953	507	16.39	10.38	
c.EU1	3L	13665384	13696922	507	16.08	4.73	
c.EU2	3L	10436331	10471601	505	14.32	7.98	
c.EU3	2L	5938111	5970628	508	15.62	13.62	
