# Supplementary material for: Pericentromeric heterochromatin is hierarchically organized and spatially contacts H3K9me2 islands in euchromatin
Source: PLoS Genet. 2020 Mar 23;16(3):e1008673. doi: 10.1371/journal.pgen.1008673 (PMC7147806; doi:10.1371/journal.pgen.1008673)
Supplement: S6 Table — (PDF) [file pgen.1008673.s026.pdf]

**S6 Table. Properties of euchromatic TEs interacting with PCH**

| | 3D interaction criteria | | | |
|---|---|---|---|---|
| | **both rep. sig.** ***p-values*** | **either rep.sig** ***p-values*** | **test type** | **Note** |
| Distance to centromere | **2.69E-02** | **3.73E-02** | *Mann-Whitney U* | with 3D interaction shorter |
| X vs autosome | 1.00E+00 | 1.00E+00 | *Fisher's Exact* | |
| extent of H3K9me2 spread | 1.47E-01 | 3.03E-01 | *Mann-Whitney U* | |
| % increase in H3K9me2 enrichment | 1.90E-01 | 5.34E-01 | *Mann-Whitney U* | |
| type of TEs (TIR, non-LTR, LTR) | 5.06E-01 | 9.84E-02 | *Chi-square* | |
| DNA vs RNA TEs | 6.33E-01 | 2.39E-01 | *Fisher's Exact* | |
| Population frequencies | **1.03E-02** | **4.19E-03** | *Student t test* | with 3D interaction lower |
