# Supplementary material for: Pericentromeric heterochromatin is hierarchically organized and spatially contacts H3K9me2 islands in euchromatin
Source: PLoS Genet. 2020 Mar 23;16(3):e1008673. doi: 10.1371/journal.pgen.1008673 (PMC7147806; doi:10.1371/journal.pgen.1008673)

S1 Text: Trans epigenetic effects of TEs

We proposed that the spatial interactions between euchromatic loci and PCH on one chromosome could influence the chromatin environment of the homolog, such as *trans*-silencing, due to strong somatic pairing in *Drosophila* (see main text). To address this question, we focused on polymorphic TE insertions between genomes and investigate whether they could lead to *trans*-epigenetic effects, or influencing the chromatin of the homolog, when in heterozygous states. We analyzed the enrichment of H3K9me2 around heterozygous TE insertions in two wildtype strains (RAL315 and RAL360) and their F1 offspring (from both directions of the cross). Maternal and paternal alleles were distinguished using previously identified SNPs in the two strains [1]. We modified the approach in [2], which compares the H3K9me2 enrichment level of *all sites* in a window between strains with and without TE insertions. Instead, we only compared the H3K9me2 enrichment level of *SNPs,* and the *trans* effects would be confirmed if the H3K9me2 fold enrichment levels for both allelic states of a *SNP* in the F1 (with and without TEs) are higher than in the parental strain without the TE.

Eight and 17 TEs have significant signals of *trans*-epigenetic effects in either direction of the crosses, which accounted for 7.9% and 16.8% of all TEs analyzed. **S1 Text – S1 Fig (left)** shows an exemplar TE with *trans*-epigenetic effects. While H3K9me2 enrichment is only found in the parental strain with the TE insertion, such enrichment was found for *both* alleles, with and without TE insertion, in the F1 offspring. In contrast, **S1 Text – S1 Fig (right)** shows an exemplar TE without *trans*-epigenetic effects. Intriguingly, we did not find TEs with significant *trans*-epigenetic effects for both direction of the cross. However, given the high false negative rates of analysis based on SNP alone (see below), it is still inconclusive if there are strong maternal effects for the observed *trans*-epigenetic effects. Unlike TEs identified as spatially interacting with PCH (see main text), we did not find differences in the distance to PCH between TEs with and without *trans*-epigenetic effects. Many factors could have contributed to this. For example, the strength of homolog pairing varies across the genome [3], which could also influence the tendency of a TE to show *trans*-epigenetic effects.

It is worth noting that there is on average ~20 SNPs within 1kb window, which suggests lower statistical power for analysis based on *SNPs* alone than that based on *all sites*. SNPs that are within 100bp are likely covered by the same sequencing read and thus contain redundant information for our inference. Random down sampling SNPs that are within 100bp led to even fewer SNPs with unique information in a 1kb window (~5). Indeed, we found a high false negative rate (46.29%) by comparing results based on *SNP-based* and *all-sites* approach for identifying TE-induced H3K9me2 enrichment in the *parental strain*. Future analysis involving more strains that have larger genetic divergence, coupled with Hi-C studies and homolog pairing maps, will help further address the potential functional consequence of euchromatin-PCH 3D contacts.

Methods

We performed ChIP-seq targeting H3K9me2 using 16-18hr embryos of RAL315, RAL360, and the F1 of the two wildtype strains. TE insertion positions in these two strains

were from [4] and we only included TEs with coverage ratio at least three, which is a highly stringent threshold.

We downloaded genomes of RAL315 and RAL360 from Drosophila Genome Nexus (in release 5, [5]) and followed the recommendations by masking following regions that are error-prone: 3bp around indels, regions of identity by descent, and regions with residual heterozygosity. After such filtering, we generated a list of SNP that have different allelic states between RAL315 and RAL360. Raw reads from ChIP-seq experiments were pre-processed as methods described in main text. Filtered reads were mapped to *combined genomes of RAL315 and RAL360* using bwa mem with default parameters. We generated pileup tables, which contain counts for alternative alleles, using samtools' mpileup command with mapping quality cutoff 20. We filtered out SNPs whose alternative allele (e.g. RAL360 allele in RAL315) has more than one supporting reads *in the parental strains* (i.e. potentially heterozygous SNPs in parental strains). For F1s, we only included SNPs whose combined read depth of two replicates were above three.

To estimate allelic-specific fold enrichment of H3K9me2 for each SNP, we first (1) normalized sequencing depth between IP and INPUT samples using total read depth and (2) divided the normalized read count of IP by normalized read count of INPUT sample. Because the background H3K9me2 enrichment levels are different between genotypes, we used median fold enrichment of *SNPs* in +/-20-40kb to normalize the background enrichment levels between genotypes. We used one-side *Mann-Whitney U test* to investigate whether the fold enrichment of *SNPs* in (1) the parental strain with TE, (2) with TE allele in F1 or (3) without TE allele in F1 is higher than that in the parental strain without TE (**S1 Text – S2 Fig**). A TE is deemed as having *trans*-epigenetic effects if both F1 alleles with and without TEs have significantly higher H3K9me2 fold enrichment than that of the parental strain without the TE. The window size used was defined by finding the extent of H3K9me2 spread from TEs using *all-sites method* as previously described (**S1 Text – S2 Fig,** [2]). TEs that do not have H3K9me2 enrichment in the parental strain with TE or have fewer than 10 surrounding SNPs were excluded from the analysis. In total, 101 TEs were analyzed. All analysis combined RAL315-speicifc and RAL360-specific TEs together.

References:

1. Huang W, Massouras A, Inoue Y, Peiffer J, Ràmia M, Tarone AM, et al. Natural variation in genome architecture among 205 Drosophila melanogaster Genetic Reference Panel lines. Genome Res. 2014;24: 1193–1208. doi:10.1101/gr.171546.113
2. Lee YCG, Karpen GH. Pervasive epigenetic effects of Drosophila euchromatic transposable elements impact their evolution. eLife. 2017;6. doi:10.7554/eLife.25762
3. Erceg J, AlHaj Abed J, Goloborodko A, Lajoie BR, Fudenberg G, Abdennur N, et al. The genome-wide multi-layered architecture of chromosome pairing in early Drosophila embryos. Nat Commun. 2019;10: 4486. doi:10.1038/s41467-019-12211-8
4. Rahman R, Chirn G, Kanodia A, Sytnikova YA, Brembs B, Bergman CM, et al. Unique transposon landscapes are pervasive across Drosophila melanogaster genomes. Nucl Acids Res. 2015;43: 10655–10672. doi:10.1093/nar/gkv1193
5. Lack JB, Cardeno CM, Crepeau MW, Taylor W, Corbett-Detig RB, Stevens KA, et al. The Drosophila Genome Nexus: A Population Genomic Resource of 623 Drosophila melanogaster Genomes, Including 197 from a Single Ancestral Range Population. Genetics. 2015; genetics.115.174664. doi:10.1534/genetics.115.174664

S1 Text – S1 Fig. H3K9me2 enrichment level around exemplar TEs in parental strains (P) and F1. A TE on 3R (left) was found to have *trans*-epigenetic effects while another TE on the 3L (right) was not. Window size is 2kb, with LOESS smoothing (alpha = 0.05).

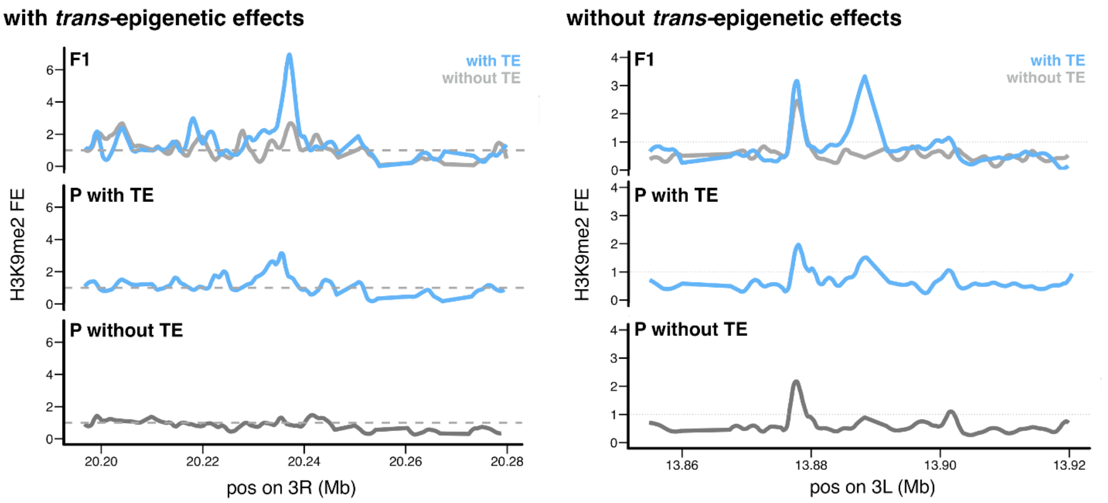

S1 text – S2 Fig. *SNP-based* and *all-sites* based method for identifying the epigenetic effects of TEs. Smoothed lines represent H3K9me2 fold enrichment across the genome (all sites), while vertical lines represent the H3K9me2 fold enrichment for SNPs.

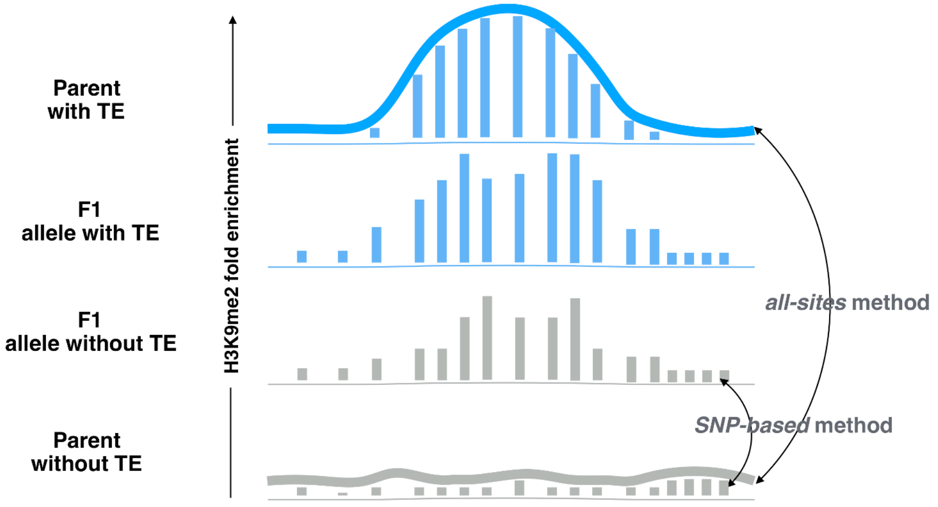

Supplement: S1 Text — (PDF) [file pgen.1008673.s027.pdf]
