# Supplementary material for: Pericentromeric heterochromatin is hierarchically organized and spatially contacts H3K9me2 islands in euchromatin
Source: PLoS Genet. 2020 Mar 23;16(3):e1008673. doi: 10.1371/journal.pgen.1008673 (PMC7147806; doi:10.1371/journal.pgen.1008673)
Supplement: Attachment — Submitted filename: Response_to_reviewers_Final.pdf [file pgen.1008673.s030.pdf]

We would like to start by thanking the reviewers for their very helpful comments. We were very pleased with the overall positive responses to our study of the spatial organization of *Drosophila* heterochromatin and its interaction with distant euchromatic loci. We appreciate the opportunity to correct our previous oversights, and to improve the descriptions of the results, methods, and conclusions. Thanks for the suggestions, we believe the revised manuscript is much improved, and hope it is now acceptable for publication in PLoS Genetics.

Reviewer 1

I have two main criticism to the microscopic experiments:

(1a) First of all, I do not understand how the authors measured the distances between FISH signals. In the figure 2C, they indicate "distances between centroids". In the M&M section they write that "Distances between foci were measured by Fiji linetool". The latter excludes the former. Linetool can be used either on projections or on single optical sections. Since the authors claim that they performed 3D analysis of the PCH and other chromosomal regions, I expect that 3D distances are calculated from measured geometrical centers (e.g., using Fiji). Was it the case?

We agree with the reviewer that our original descriptions were inaccurate and led to confusion. We took optical slices of embryos and measured the distance between centers of FISH signals on a single plane. Accordingly, some nuclei may have FISH signals for only one or neither probe, and our analysis only included those with signals from both probes. Because the same approach was used for both test and control groups, any unknown biases associated with the method should be applicable to both groups of nuclei and would not bias our results. We revised legends of Figure 2C (and other figures) and included additional details in the Materials and Methods (page 39, line 14-18) to clarify.

(1b) A related question: how the authors estimate "overlapping"? Was it actually measured or estimated by eye? And what is the "natural threshold"? (lines 19-22). I believe, the authors have to explain how the distance measurements and scoring was performed in more detail.

We used the distribution of distance between two foci to define the threshold for "overlapping." From Figure 2E, 3F, 4B, and 4D, it is apparent that the distance distribution between the two foci on a single slice is bimodal. There is a large peak near zero, which reveals a "natural threshold" dividing the nuclei into two groups. We thus defined foci with distances shorter than this threshold, effectively the peak around zero, as "overlapping." We further clarified this point in text (page 8, line 22 – page 9, line 3).

(2).1 Secondly, I do not understand why the authors prefer relative distances to absolute distances. Interactions between foci, especially in case of liquid-liquid phase separation, are physical interactions, they either exist or do not exist, and whether they occur in a small or large nucleus is not important. Besides, as I can judge from images in the figures, the differences in the nuclear sizes are pretty small.

We originally presented analysis based on relative distance because other studies using cell culture found changes in nuclear volume upon hexanediol treatment. However, as the reviewer observed, we did not see a significant change in nuclear size between embryos with and without hexanediol treatment. Also, we agree with the reviewer that liquid-liquid phase separation is physical interactions and absolute distance would be more appropriate. Accordingly, we instead presented analysis based on absolute distance in the main text. We now include the comparison of

nuclear size between embryos with and without hexanediol treatment (Figure S12; page 22, line 1-3) and analysis based on relative distance (Figure S11; page 22, line 3-5) in the supplementary materials.

(2).2 Moreover, it seems to me that nuclear radii were defined arbitrary, probably using the Fiji linetool. For proper measurements of a nuclear radius, one would need to segment a nucleus and define its geometrical center, on a first place. Although in M&M part (lines 11-18) there is no indication of how the radii ware measured, I do not think the authors performed such measurements.
We agree with the reviewer that our original descriptions were unclear. Since all the images are optical slices, we also used Fiji linetool to measure the radius of DAPI region on the section. We revised Materials and Methods accordingly (page 39, line 14-17).

Minor comments:
(3a) All microscopic figures: According to a good publishing practice, all single channel panels have to be presented as grey scale images, while leaving RGB panels only for overlays of false colored channels.
We appreciate reviewer pointing this out, and we have corrected images appropriately.

(3a) All microscopic figures: The authors should indicate whether presented images are single confocal sections or projections. If projections, they should indicate of how many sections (distances).
All the images in this study are optical sections of embryos. We revised all relevant Figure legends accordingly.

(3a) All microscopic figures have no scale bars.
We have added scale bars.

(4) Figure 4: Why there is such a big difference between TE1-PCH overlapping frequency on graph 4B and 4D, 17.2% and 7.7%, respectively?
We agree with the reviewer that this is a puzzling result, which we believe arises from differences in embryo permeabilization and fixation procedures in 4B and 4D. Embryos in 4B were permeabilized and fixed at the same time (using heptane and formaldehyde (in PBS) biphasic solution). On the other hand, in order to test the effect of 1,6-hexanediol on live embryos, embryos in 4D were first permeabilized using a combination of d-limonene and surfactants (termed "EPS"), treated with 1,6-hexanediol, and then fixed. The surfactants, basically detergent, also have the potential to perturb hydrophobic interactions and could thus lead to fewer overlapping foci between TE1 and PCH even in the control embryos (embryos with EPS but not 1,6-hexanediol treatment). However, this is a hypothesis, so we refrained from raising this point in the manuscript. We can do so if the reviewer thinks it is necessary.

It is worth noting that to accommodate any potential influence of different procedures of fixations, we compared embryos with 1,6-hexanediol treatment (treated with EPS first) only to control embryos with EPS treatments.

(5) On some of DAPI nuclear images (when converted to grey scale), one can see not a single chromocenter but two or at least a bipartite structures intensely stained with DAPI. I am not familiar with Drosophila nuclei, but could it be that a chromocenter is split in two? How has this influenced

measurements? The authors have to mention this point.
Indeed, the number of chromocenters in *Drosophila* nuclei varies between one and two. When there is more than one chromocenter, which is reflected by more than one AAGAG focus, we measured the distance between Oligopaint focus and the nearest AAGAG focus. Because we did not find difference in the number of AAGAG foci between test and control groups (Hi-C strain vs wildtype strain; EPS treatment vs EPS+1,6-hexanediol treatment), this varying number of chromocenters should not influence our analysis. We further revised the section in Materials and Methods (page 39, line 18 – page 40, line 2).

(6a) The authors do not discuss what causes the hierarchy in the chromocenter structure with PCH. It seems that PCH on X chromosomes is relatively distanced from other PCHs. Could it be because the X-chromosome in Drosophila harbors NOR and the formed nucleolus might impose some geometrical constrains?
We speculated that the hierarchical organization of PCH could be due to non-random positioning of PCH regions upon mitotic exit or varying biophysical properties of PCH. We agree with the reviewer this (structural constrain) could be a potential possibility and included additional discussions (page 26, line 10-15).

(6b) Can one explain the different degree of clustering or chromosomes 2 and 3 with entirely heterochromatic chr.4 by different amount of PCH on these chromosomes?
This is an interesting point. Indeed, *in our Hi-C analysis,* there are more *unique* heterochromatic reads for 3rd PCH (39.67%), 2nd PCH (35.74%), and 4th chromosome (12.61%) than PCH of other chromosomes. However, these estimates are limited to single-copy heterochromatic regions. Accordingly, we are unable to test whether the size of heterochromatin (consisting of both single-copy and multi-copy sequences) is responsible for this observation.

(7) I am missing a discussion about the phenomenon of contacts between "H3K9me2 islands" and PCH. Do authors explain such contacts as a mechanism of genome regulation or simply as an inevitable consequence of liquid-liquid phase separation caused by HP1 enrichment in both loci? If it is important for transcription regulation, is similar phenomena exist in nuclei with polytene chromosomes?
We agree with the reviewer that both could be possible. In addition to our original discussion speculating that 3D interaction between H3K9me2 islands and PCH is an inevitable consequence of liquid-liquid phase separation, we included new discussion on the possible role of this spatial interaction in transcription regulation (page 25, line 20 – page 26, line 7).

(8) The authors need to specify which age of D.melanogaster embryos was used by Schuettengruber et al (2014) – in their paper I found only indication that ChIP experiments on whole Drosophila embryos were performed 4–12 hr after egg laying, which is different from the age of embryos used by Lee at al (16-18 hr)
Schuettengruber et al (2014) data were generated from 16-18hr D. melanogaster embryos (please see NCBI GEO report https://www.ncbi.nlm.nih.gov/geo/query/acc.cgi?acc=GSM1505557). This is also the stage we used to perform all other experiments. We included this information in page 6, line 18.

(9) In M&M, there is no information about how confocal stacks were acquired (voxel size, lasers,

etc) and whether axial chromatic shift correction was performed
Our image analyses were performed on single slices; we now make this clear in Materials and Methods (page 39, line 14-16).

(10) Number of Supplementary figures is unnecessarily high and should be reduced, for instance, figures S4 and S5 can be joined; the same is for figures S13 and S14.
We agree with the reviewer and have reduced the number of S figures (from 25 to 20) through fusion.

(11) The left part of the schematics in Figure 5, in my view, can be omitted
We would like to keep the left view in order to emphasize that TEs may be able to contribute to variation in 3D genome structure.

Reviewer 2
1. Chromosome 4 shows more interactions with other regions of heterochromatin than expected despite its small size. This observation raises the question of what might be the cause. In the model figure, chromosome 4 is drawn at the center of the heterchromatic compartment/chromocenter, which would explain this observation. Is there data from the FISH experiments that support this part of the model?
Our model is inferred from our Hi-C data and Rosin et al. 2018 PLoS Genetics. Rosin et al. used Oligopaint labeling of the entire euchromatic genome to establish the spatial positions of five euchromatic arms, based on which we inferred the spatial positions of PCH of corresponding chromosomes. Because in our Hi-C analysis, 4th chromosomes show the strongest interaction with 3L- and 3R-PCH, we placed 4th chromosome in the middle of chromocenter. Alternative configurations placing 4th chromosome outside would be inconsistent with our Hi-C data. Our FISH analysis was unable to label all PCHs and 4th chromosome simultaneously and thus unable to provide direct support for the model. In the legend of Figure 5, we provided additional details of how this model is generated.

We included new discussion on the potential cause for varying spatial interactions between 4th and other PCHs in page 26, line 15-21. In short, the unique chromatin environment of the 4th chromosome could influence its biophysical properties and thus propensity of the chromosome to liquid-liquid fuse with other PCHs.

2. Among the euchromatic H3K9me2/3 regions, only a small fraction, ~10%, actually contact the pericentric heterochromatin. This number is similar for regions including TEs and those H3K9me2/me3 enriched regions lacking TEs. Other than proximity on the linear chromosome, what predicts if an H3K9me2/me3 enriched region contacts the PHC?
In addition to the proximity to PCH on a linear chromosome, we also tested the effects of sex chromosome linkage, size of the H3K9me2 enriched region, level of H3K9me2 enrichment, TE family class or type. However, none of these properties significantly associate with whether a region spatially interacts with PCH or not. These analyses are reported in page 14, line 12-16 and Table S4 (H3K9me2 islands) and page 18, line 4-6 and Table S6 (TEs).

3. The H3K9me2/me3 enriched regions associated with the PHC are reported to be associated with open/active chromatin states. However, the chromatin state data is from a different chromatin source. Is there information available regarding the expression status of the genes within the H3K9me2/me3 enriched regions? The expectation would be that they should be transcriptionally silent.
We agree with the reviewer that the chromatin datasets (5 colors or 9 states) are of a different source (cell lines) from our dataset (16-18hr embryos). We thus used modEncode 16-18hr embryo transcriptome data to further investigate whether genes in H3K9me2 islands interacting with PCH have suppressed expression. Surprisingly, compared to other genes in H3K9me2 islands without PCH interactions, these genes actually have *higher* expression, which is consistent with the analysis based on chromatin. We included this new analysis in page 14, line 4-9.

While this result is intriguing, we also noted in the main text that "since the chromatin states and expression data analyzed are from strains that have different H3K9me2/3 distributions from those of the Hi-C strain, additional studies are needed." (page 14, line 10-12).

4. Also, if the hypothesis is that the H3K9me2/me3 enriched regions with TEs associated with PHC are selected against due to the impact on gene expression, is this idea supported when examining gene expression from 16-18hr embryos? While not ideal due to genotype differences, it might be possible to use modENCODE data to address this question.
We appreciate the great suggestion by the reviewer. However, the ORw1118 strain used in the study is generated by introgressing *white* into Oregon-R background. Accordingly, the genetic background and distribution of TE insertions of ORw1118 and Oregon-R, from which the modEncode data were generated, are different. TE insertions and the associated H3K9me2 islands studied in ORw1118 are thus unlikely to be present in Oregon-R, preventing us from testing the expressional effect of TEs associated with PCH. It is worth noting that even slight changes in expression level detected in the laboratory setting could have large fitness consequences in nature. Future transcriptomic studies of the ORw1118 strain and multiple wildtype strains will hopefully provide enough statistical power to detect differences in expression levels of alleles with and without PCH-associating TEs, which would help address this question.

5. Regarding the hypothesis that TE-regions interacting with the PHC are selected again, an alternative explanation might be a difference in age of the TE insertions between PHC-associated and other TE-H3K9me2/me3 regions. Sequence data from the TEs in question might be available to test this idea.
This is an excellent question raised by the reviewer. Unfortunately, the sequencing technology we used for genome sequencing (Illumina pair-end) only allows identification of the "location" of TE insertions, but not "sequences" for individual TE insertions. Accordingly, we are unable to test whether TE insertions that show PCH interactions are younger/older than other copies of the same TE family.

Reviewer 3:
Major comments:
1. The justification for the "unique", "repeat", and "multi" categories was unclear to me. Why did the authors separate reads from multicopy DNA into "repeat" reads and "multi" reads into two

categories? How did this partitioning affect their results?
We agree with the reviewer that both "repeat" and "multi" categories are from multicopy DNA, and, based on our original text, it is difficult to understand why we have this partition. We have two categories mainly for technical reasons. The "repeat" category consists of simple and complex repeats already known to be enriched in heterochromatin, but are not necessarily fully assembled/included in the genome sequence. To identify this category of reads, instead of aligning reads to the assembled genome sequence, we aligned reads to a list of known heterochromatic repeats. The "multi" reads are reads that can be aligned to assembled heterochromatic genome. However, unlike "unique" reads that only mapped to one location in the assembled heterochromatic genome, these "multi" reads mapped to multiple locations.

To clarify this point, we revised text in Results (page 6, line 9-15) and Materials and Methods (page 33, line 22-page 34, line 7).

2. Do the euchromatic H3K9me2/3 islands interact with other euchromatic H3K9me2/3 islands on the same chromosome? If all of the islands interact with their own PCH, then you would expect this to be the case, but these interactions may be hierarchical even within an arm. If so, intra-arm Eu H3K9me2/3 interactions (compared to inter-arm Eu H3K9me2/3 interactions) might be stronger and easier to see than PCH-Eu interactions.
This is a very interesting question, but cannot be answered with our current Hi-C data.
We found very few (<10) Hi-C read pairs supporting the interactions between pairs of euchromatic H3K9me2 islands (as compared to > 1000 Hi-C read pairs for pairs of H3K9me2-PCH interactions). However, this observation does not necessarily suggest the lack of spatial interactions between them. The sizes of H3K9me2 islands are much smaller than PCH (several kb vs several Mb). This would proportionally lead to few observed Hi-C read pairs even for H3K9me2 islands that do interact spatially. This lack of information in our current dataset thus makes it difficult to identify interacting H3K9me2 islands. In addition, while we know the exact size of H3K9me2 islands, the size of PCH is still unknown due to the unassembled heterochromatic repeats. This makes it difficult to normalize and compare the observed number of Hi-C read pairs supporting either interactions among H3K9me2 islands or interactions between H3K9me2 islands and PCH. Future analysis with even more deeply sequenced Hi-C data and/or multi-color FISH simultaneously labeling multiple euchromatic H3K9me2 islands and PCHs of different chromosomes could potentially address this interesting question.

3. The authors showed that euchromatin-PCH interaction is sensitive to perturbing liquid-liquid phase separation. Did they confirm that the PCH-PCH domain interactions are also perturbed in their experiments?
We agree that this is an interesting question. Our experiment could not answer this question directly because the probes used in the experiment with hexanediol treatment could not distinguish between PCH of different chromosomes. Nevertheless, PCH-PCH interactions are likely also perturbed with hexanediol treatment given the dispersion of the whole HP1a domain in S2 cells upon 1,6-hexanediol treatment (Strom et al. 2017 Nature).

4. The motivation for the experiments described on page 18 and in figure 4 was not clear. The result is interesting, but may require reframing. The authors say that polymorphic TEs allowed them to determine the effect of TE-induced H3K9me on euchromatin-PCH interactions. However, the control

TEs (c.TE1 and c.TE2) are those without H3K9me2/3 enrichment and without interaction with PCH, but they are also much further away from the PCH on the linear chromosome than the polymorphic TEs (TE1 and TE2) that interact with the PCH and have H3K9me enrichment. It seems that all that can be concluded from this experiment is that a transposable element alone is not sufficient to cause an interaction with the PCH. Perhaps a better control for asking about the effect of TE-induced H3K9me2/3 on interaction with PCH is to compare these TEs with a pair equally close to the PCH but without H3K9 methylation

We agree with the reviewer that our original description was unclear and caused confusion. It is difficult, if not impossible, to have test and control regions that are of equal distance to PCH, which is the limitation of our FISH analysis on TE-free H3K9me2 islands (discussed in page 15, line 17-21).

In contrast, polymorphic TEs offered the rare opportunity to compare the 3D structures of *homologous sequences* with and without TEs. In other words, we could compare the same sequences in the same genomic context that differ in the presence/absence of a TE insertion. Accordingly, instead of comparing between tested TEs (TE1 and TEs) and control TEs (c.TE1 and c.TE2), our analysis focused on comparing the 3D structures of TE-neighborhood between strains *with* and *without* these TEs. We added additional descriptions and hope this clarified the point (page 18, line 12-19).

Minor:

5. Page 24 line 20: "PHC" should be "PCH"

We thank reviewer for pointing out this typo and we have fixed it.

6. Page 37 lines 12, 15, 18: fix ug and uL character

We thank reviewer for pointing out these typos and we have fixed them.

7. Fig 3F: threshold for overlapping might work better as a dotted line through each plot than an arrow on the first plot

We appreciate reviewer's suggestion and have changed that for Figure 3F, Figure 4B and Figure 4D.
