# Supplementary material for: Pericentromeric heterochromatin is hierarchically organized and spatially contacts H3K9me2 islands in euchromatin
Source: PLoS Genet. 2020 Mar 23;16(3):e1008673. doi: 10.1371/journal.pgen.1008673 (PMC7147806; doi:10.1371/journal.pgen.1008673)
Supplement: Attachment — Submitted filename: Review_112019.docx [file pgen.1008673.s029.docx]

The manuscript entitled “Pericentromeric heterochromatin is hierarchically organized and spatially contacts H3K9me2 islands in euchromatin” by Lee and colleagues describes the latest results of the continued efforts by the Karpen laboratory to understand the organization of the heterochromatin in *Drosophila melanogaster*. They present the first in-depth analysis of the 3D structure of *D. m.* pericentric heterochromatin and its interaction with euchromatic domains. Utilizing a new methods in combination with cytogenetics analyses, they find that euchromatic H3K9me2/me3-enriched domains preferentially interact with pericentric heterochromatin, likely by participating in liquid-liquid phase-separated domains.

The manuscript is well written and easily accessible to the reader. The figures are well laid out, the figure legends include the necessary information, and the data shown support the conclusions drawn by the authors.

The following questions arose during the review of the manuscript, which the authors are encouraged to address in a revised manuscript.

- Chromosome 4 shows more interactions with other regions of heterochromatin than expected despite its small size. This observation raises the question of what might be the cause. In the model figure, chromosome 4 is drawn at the center of the heterchromatic compartment/chromocenter, which would explain this observation. Is there data from the FISH experiments that support this part of the model?
- Among the euchromatic H3K9me2/3 regions, only a small fraction, ~10%, actually contact the pericentric heterochromatin. This number is similar for regions including TEs and those H3K9me2/me3 enriched regions lacking TEs. Other than proximity on the linear chromosome, what predicts if an H3K9me2/me3 enriched region contacts the PHC?
- The H3K9me2/me3 enriched regions associated with the PHC are reported to be associated with open/active chromatin states. However, the chromatin state data is from a different chromatin source. Is there information available regarding the expression status of the genes within the H3K9me2/me3 enriched regions? The expectation would be that they should be transcriptionally silent.
- Also, if the hypothesis is that the H3K9me2/me3 enriched regions with TEs associated with PHC are selected against due to the impact on gene expression, is this idea supported when examining gene expression from 16-18hr embryos? While not ideal due to genotype differences, it might be possible to use modENCODE data to address this question.
- Regarding the hypothesis that TE-regions interacting with the PHC are selected again, an alternative explanation might be a difference in age of the TE insertions between PHC-associated and other TE-H3K9me2/me3 regions. Sequence data from the TEs in question might be available to test this idea.
